# Synthesis of tetra- and octa-aurated heteroaryl complexes towards probing aromatic indoliums

**DOI:** 10.1038/ncomms11489

**Published:** 2016-05-17

**Authors:** Jun Yuan, Tingting Sun, Xin He, Ke An, Jun Zhu, Liang Zhao

**Affiliations:** 1 Key Laboratory of Bioorganic Phosphorus Chemistry and Chemical Biology (Ministry of Education), Department of Chemistry, Tsinghua University, Beijing 100084, China; 2 State Key Laboratory of Physical Chemistry of Solid Surfaces and Collaborative Innovation Center of Chemistry for Energy Materials (iChEM), Fujian Provincial Key Laboratory of Theoretical and Computational Chemistry and Department of Chemistry, College of Chemistry and Chemical Engineering, Xiamen University, Xiamen 361005, China

## Abstract

Polymetalated aromatic compounds are particularly challenging synthetic goals because of the limited thermodynamic stability of polyanionic species arising from strong electrostatic repulsion between adjacent carbanionic sites. Here we describe a facile synthesis of two polyaurated complexes including a tetra-aurated indole and an octa-aurated benzodipyrrole. The imido trinuclear gold(I) moiety exhibits nucleophilicity and undergoes an intramolecular attack on a gold(I)-activated ethynyl to generate polyanionic heteroaryl species. Their computed magnetic properties reveal the aromatic character in the five-membered ring. The incorporation of the aurated substituents at the nitrogen atom can convert non-aromaticity in the parent indolium into aromaticity in the aurated one because of hyperconjugation. Thus, the concept of hyperconjugative aromaticity is extended to heterocycles with transition metal substituents. More importantly, further analysis indicates that the aurated substituents can perform better than traditional main-group substituents. This work highlights the difference in aromaticity between polymetalated aryls and their organic prototypes.

## Abstract (web summary)

Polymetalated aromatic compounds are challenging synthetic goals. Here, the authors describe the synthesis of tetra-aurated indole and octa-aurated benzodipyrrole complexes, in which incorporation of the aurated substituents at the nitrogen atoms leads to hyperconjugative aromaticity in the five-membered rings.

## Graphical abstract

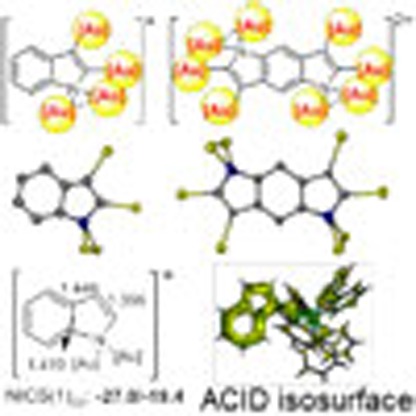

Aryl- and heteroaryl-Au(I) compounds have recently attracted substantial attention because of their crucial intermediate role in versatile gold-catalysed transformations for the formation of new carbon–carbon and carbon–heteroatom bonds^1^,^2^,^3^,^4^,^5^. Structural characterization and reactivity studies of the organogold intermediates facilitated comprehension of the reaction mechanisms and provided inspiration for developing new synthetic methodologies^6^,^7^,^8^,^9^. Moreover, great interest has also arisen in the exploration of rich reactivity of aryl-Au(I) compounds. They can serve as an organometallic partner for metal-catalysed (for example, Pd and Ni) cross-coupling reactions^10^, as a nucleophile to react with diverse appropriate electrophiles^11^, and as a key intermediate to undergo a Au(I/III)-catalysed C-H activation and cross-coupling reaction in the presence of oxidants^12^,^13^. In addition, due to the attractive aurophilic interaction^14^,^15^ the d^10^ gold(I) atom holds a great potential to aggregate into a polynuclear cluster. This structural characteristic perplexes the mechanism studies of gold-catalysed reactions, especially when bi- or polynuclear gold catalysts were used^16^,^17^. Therefore, the structure and reactivity studies of polyaurated organometallics may not only lead to new reagents for use in synthesis but also help understand the reaction mechanisms of Au-catalysed reactions. Furthermore, detailed structural characterization of polymetalated aryls may also provide a new insight into our current ideas about bonding between metal ions and organic species. However, relative to numerous reported mono-metalated aryls^18^,^19^,^20^ derived from direct electrophilic metalation or halogen-lithium-metal exchange reactions^21^, to date polymetalated aryl compounds still represent a very rare class of molecules^22^,^23^ that are synthetically hard to reach by conventional methods. The formidable challenge of this synthesis lies in the difficult generation of multi-topic anions and the limited thermodynamic stability of polyanionic species due to strong electrostatic repulsion between adjacent carbanionic sites.

On the other hand, the concept of hyperconjugative aromaticity was first proposed in 1939 by Mulliken^24^, who considered that the saturated CH_2_ group in cyclopentadiene could contribute ‘pseudo' 2*π* electrons by hyperconjugation to the four olefinic *π* electrons, thus leading to aromatic cyclopentadiene with 6π electrons. Later, Schleyer, Nyulászi and O'Ferrall extended this concept and found that electropositive substituents (for example, SiH_3_, GeH_3_ and SnH_3_) led to enhanced hyperconjugative aromaticity whereas electronegative substituents (for example, F, Cl) resulted in anti-aromatic five-membered rings^25^,^26^,^27^. Very recently, Houk revealed that the transition state distortion energies for the Diels–Alder reactions of 5-substituted cyclopentadienes with ethylene or maleic anhydride were directly related to the hyperconjugative aromaticity^28^. We found that hyperconjugation could play an important role in triplet state aromaticity^29^. However, all the previous studies on hyperconjugative aromaticity are mainly limited to carbocycles and all the substituents considered are main-group elements only.

In this contribution, we disclose a facile synthesis of two cationic polymetalated heteroaryl compounds including a tetra-aurated indolium and an octa-aurated benzodipyrrole complex. We find that the μ_3_-imido aurated species exhibit good nucleophilicity and undergo an intramolecular attack on a Au(I)-activated ethynyl to generate polyanionic heteroaryls. The polyaurated heteroaryls exhibit reasonable stability upon exposure to air. The theoretical investigation reveals that the incorporation of the aurated substituents at the nitrogen atom can not only convert non-aromaticity in the parent indolium into aromaticity in aurated one due to the hyperconjugation but also lead to the most aromatic indolium. Thus, our findings not only extend the concept of hyperconjugative aromaticity to transition metal-involved heterocycles and accounts for the excellent stability of two polyaurated heteroaryls but also reveal better performance of transition metal-involved substituents over the traditional main-group ones on hyperconjugative aromaticity. In contrast to metallabenzene complexes where a metal atom is incorporated directly into a ring^30^,^31^,^32^, the polymetalated heteroaryl compounds reported in this work represent a class of metalla-aromatics where the aromaticity is introduced by the hyperconjugation of transition metal substituents.

## Results

### Synthesis of tetra- and octa-aurated heteroaryl complexes

It is well-known that the gold(I) centre can act as an excellent soft Lewis acid to activate carbon–carbon multiple bonds. Many mononuclear organogold(I) compounds have been synthesized by the reactions of allene/alkene/alkyne substrates and nucleophiles with stoichiometric gold(I) compounds^18^,^19^,^20^,^33^. For example, *σ*- and *π*-activation of the ethynyl group of *o*-alkynylanilines by a gold(I) species led to the formation of 2- and 3-aurated indole complexes, respectively^34^,^35^. In view of the acknowledged isolobality of the proton and the LAu^+^ fragment (L=phosphine or carbene ligands)^36^, we therefore assume that an aurated imido species in an *o*-alkynylaniline derivative may act like an amine group to undergo a similar annulation reaction to react with the *π*- and/or *σ*-activated ethynyl group.

**Figure 1:**
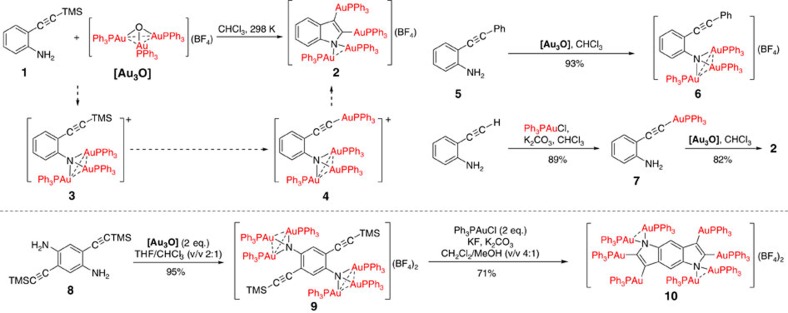
Synthesis of tetra-aurated indole 2 and octa-aurated benzodipyyrole 10. The structures of complexes **2**, **6**, **9** and **10** have been determined by X-ray crystallography.

Sharp and co-workers have previously reported that a primary amine group readily reacts with the μ_3_-oxo trinuclear gold(I) compound [(PPh_3_Au)_3_(μ_3_-O)](BF_4_) (**[Au**_**3**_**O]**) to form a μ_3_-imido trinuclear gold(I) compound^37^,^38^. According to this synthesis, we then carried out the reaction of **[Au**_**3**_**O]** (1 equiv.) with *o*-(trimethylsilylethynyl)aniline **1** (trimethylsilyl=TMS) aiming at the synthesis of the gold(I) imido complex **3** (Fig. 1). The reaction mixture experienced a colour change from light brown to yellow. Diffusion of diethyl ether to the resulting yellow solution yielded colourless crystals of complex **2**. Structural analysis by X-ray crystallography (*vide infra*) revealed that **2** has an unexpected tetra-aurated indolyl structure instead of the NAu_3_ cluster aggregate. This unusual transformation can be rationalized in the light of the following experimental facts, and divided as two steps: the auration of the amino group in **1** and the subsequent nucleophilic attack of the aurated imido on an Au(I)-activated carbon–carbon triple bond.

The electrospray ionization mass spectroscopy (ESI-MS) analysis of the reaction mixture of **1** and **[Au**_**3**_**O]** revealed the strongest signal at *m/z*=1564.2502 that can be ascribed to the 1+ species of **3** (∼1564.2548), substantiating that the μ_3_-imido trinuclear gold(I) species **3** indeed forms in the **1**-to-**2** transformation process (Supplementary Fig. 1). In order to clarify what kind of activation (*σ*-, *π*- or *σ*,*π*-mode)^39^ involved in the **1**-to-**2** transformation, we employed the inner alkyne **5** to react with **[Au**_**3**_**O]** based on the synthetic procedure for **2**. The μ_3_-imido trinuclear gold(I) complex **6** was obtained as a crystalline solid in almost quantitative yield (crystal structure is shown in Supplementary Fig. 2). No cyclization products were found in this transformation. The obtaining of **6** not only provides further evidence for the formation of the NAu_3_ species but also suggests the necessity of the *σ*-activation in the **1**-to-**2** transformation. Since the reaction of **1** and **[Au**_**3**_**O]** is too rapid to isolate the *σ*-activated complex **4**, we next conducted the reaction between a simplified analogue PhC≡CTMS and **[Au**_**3**_**O]**. The ^1^H-NMR monitoring explored the formation of the known complex PhC≡CAuPPh_3_ in the reaction mixture, suggesting that **[Au**_**3**_**O]** can break the C–Si bond in the TMS-C≡C moiety and engender the formation of a *σ*-bonded gold acetylide. This result affords a rationale for the *σ*-activation of **1** in the **1**-to-**2** transformation that should include the **[Au**_**3**_**O]**-induced C–Si bond cleavage as well. In addition, we found the *σ*-aurated complex **7**, which derived from the reaction of 2-ethynylaniline with PPh_3_AuCl, can react with **[Au**_**3**_**O]** to produce **2** quantitatively within 5 min as evidenced in the ^1^H-NMR monitoring (Supplementary Fig. 3). The **7**-to-**2** transformation supports the necessity of the *σ*-activation and meanwhile embodies the remarkable nucleophilicity of the aurated imido moiety.

We then carried out the reaction of the ditopic starting material **8** with **[Au**_**3**_**O]** to purposefully synthesize the polyaurated benzodipyrrole complex **10** based on the synthetic procedure for complex **2** (Fig. 1). However, this reaction gave a mixture of several gold-containing species which were observed in NMR spectroscopy and ESI-MS analysis. This outcome may result from several incomplete transformations including the auration of NH_2_, the σ-metalation of TMS-C≡C and the cyclization between the imido and the ethynyl group as similar as the **1**-to-**2** transformation. Fortunately, in a mixed solvent of tetrahydrofuran (THF) and chloroform (v/v=2:1) the reaction of **8** with **[Au**_**3**_**O]** (2 equiv.) produced a yellow precipitate of complex **9**. This transformation is quantitative, so further separation is not necessary. X-ray crystallographic analysis disclosed two μ_3_-imido trinuclear gold(I) moieties in the crystal structure of **9** (Supplementary Fig. 4). In view of the prerequisite *σ*-activation for the **7**-to-**2** transformation, we then conducted the reaction of **9** with Ph_3_PAuCl (2 equiv.) in the presence of potassium carbonate and fluoride in order to replace the two TMS groups by two gold(I)-containing units. Amazingly, this transformation directly resulted in the formation of the octa-aurated benzodipyrrole complex **10**, which was characterized by X-ray crystallography (*vide infra*).

**Figure 2:**
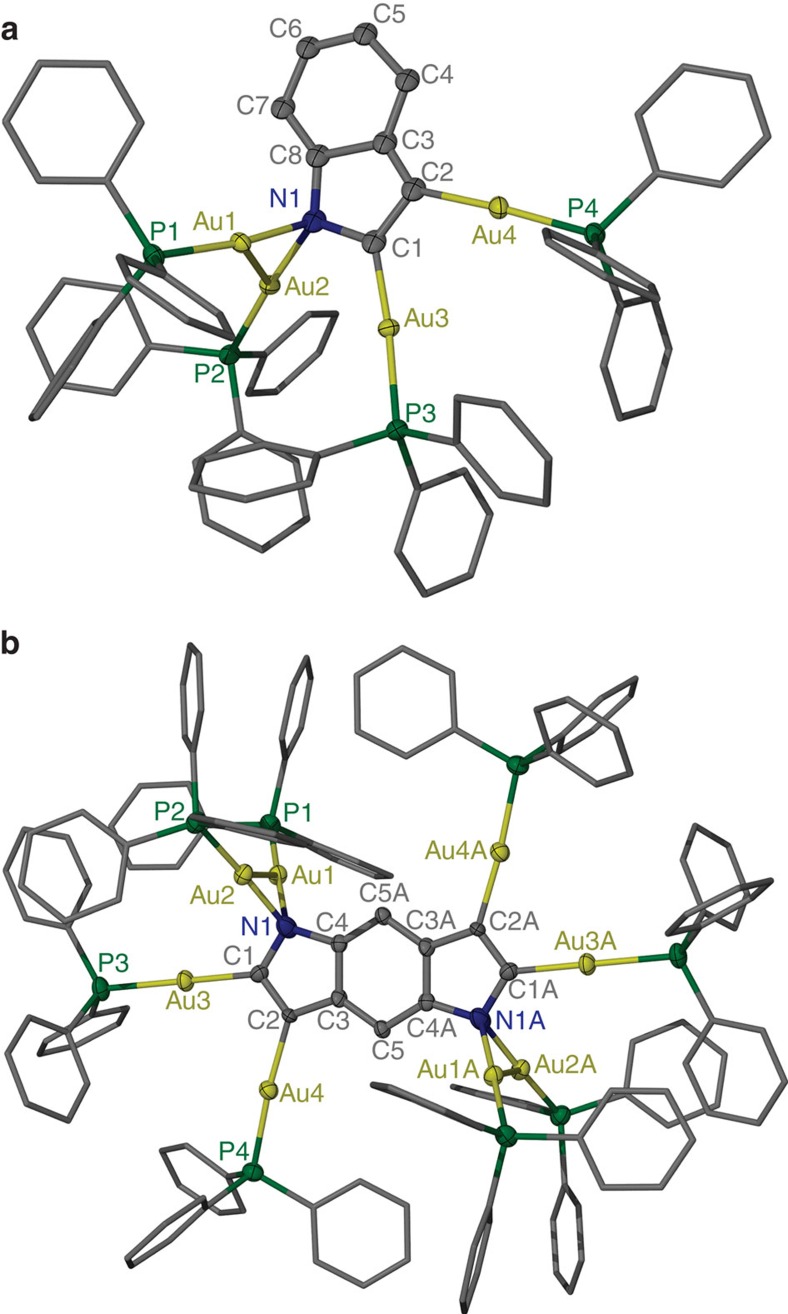
Crystal structures of heteroaryl-gold(I) complexes. (**a**) tetra-aurated complex **2** and (**b**) octa-aurated complex **10.** Hydrogen atoms and tetrafluoroborate counter anions are omitted for clarity. Dimension of the benzodipyrrolium moiety in **10** (Å): N1-C1 1.414(14); N1-C4 1.470(14); C1-C2 1.344(13); C2-C3 1.417(12); C3-C4 1.387(14); C3-C5 1.393(13); and C4-C5A 1.381(13).

Molecular structures of the tetra-aurated indole complex **2** and the octa-aurated benzodipyrrole complex **10** were unambiguously determined by X-ray crystallographic analysis and confirmed by elemental analysis, multinuclear NMR spectroscopy and ESI-MS. As shown in Fig. 2a, crystal structure of **2** is composed of a tetra-aurated indolyl trianion, four coordinative AuPPh_3_ units plus a BF_4_^−^ counter anion. The nitrogen and carbon atoms of the indolyl moiety in **2** are coplanar. Therein, carbon atoms C1 and C2 each is coordinated by a AuPPh_3_ unit with the Au–C bond lengths in the range of 2.01(3)–2.05(3) Å, comparable with the values in reported mononuclear gold(I)-indole complexes^34^,^35^. Both gold atoms (Au3 and Au4) maintain the typical linear geometry with the P–Au–C bond angles of 175.4(9)° and 175.6(7)°, respectively. The indolyl nitrogen atom N1 is bonded by two geminal gold atoms (N1-Au1=2.17 (2) Å and N1-Au2=2.08 (2) Å), which together with C1 and C8 constitute a distorted tetrahedral bonding geometry for N1. The two gold atoms are held together by a strong aurophilic interaction^14^,^15^ with the Au···Au distance of 2.862(2) Å. Although the whole structure of **2** was determined by X-ray crystallography and its composition was further substantiated by elemental analysis and ESI-MS; however, due to crystal twinning it is difficult to acquire the precise dimension of the indolyl skeleton in **2**. The mean deviation of all C–C and C–N bond lengths is as large as 0.04 Å.

Crystal structure of **10** can be described as a higher congener of **2** (Fig. 2b). To the best of our knowledge, complex **10** represents the first aurated benzodipyrrole compound. The negatively charged carbon and nitrogen atoms in the benzodipyrrole skeleton are mono- or di-aurated as similar as in **2**. An inversion centre is located in the centroid of the benzodipyrrole skeleton. Thus, two identical tetra-aurated pyrrolyl units are included in the crystal structure of **10**. The Au–C bond lengths in **10** lie in the range of 2.019(11)–2.036(8) Å, a bit shorter than the Au–N distances in the range of 2.082(11)–2.094(10) Å. The Au1···Au2 distance of 2.879(1) Å is comparable with the value in complex **2**. Although there are a number of examples containing the μ_3_-imido trinuclear gold(I) cluster^37^,^38^,^40^,^41^, the geminally diaurated fashion of a heteroaryl nitrogen atom as shown in **2** and **10** has not been reported before. In view of the isolobality of the proton and the LAu^+^ fragment^36^, such diaurated indolyl structure can be considered as an analogue of indolium. However, scrutiny of the dimension of the benzodipyrrole moiety in **10** reveals that the cyclic five-membered ring exhibits very good aromaticity with the C–N and C–C bond lengths being equalized and comparable with the parent indole.

Complexes **2** and **10** have very good stability upon exposure to air and moisture. In solution, these two complexes can also keep their structures intact as evidenced in ESI-MS and NMR. In the ESI mass spectrum of **2**, an isotopically well-resolved peak at *m/z*=1950.2641 corresponding to the [**2**−BF_4_]^+^ species was observed (Supplementary Fig. 5). The ^1^H-NMR spectrum of **2** clearly showed two multiplets at 7.12 and 7.82 p.p.m., which can be assigned to the four protons on the benzene ring of the indolyl skeleton (Supplementary Fig. 6). ESI mass spectrum of **10** revealed a strong and isotopically well-resolved peak at *m/z*=1911.7428 corresponding to the [**10** – 2BF_4_]^2+^ species (Supplementary Fig. 7). Its ^1^H-NMR spectrum gave a sharp singlet at 8.29 p.p.m. due to two isolated proton atoms on the central benzene ring of the benzodipyrrole moiety (Supplementary Fig. 8). ^31^P-NMR spectra of **2** and **10** revealed two similar resonance peaks at around 33.6 and 39.4 p.p.m. (Supplementary Fig. 9), which can be assigned to the AuPPh_3_ units attached on the nitrogen and carbon atoms.

### DFT computations on aurated heteroaryl complexes

**Figure 3:**
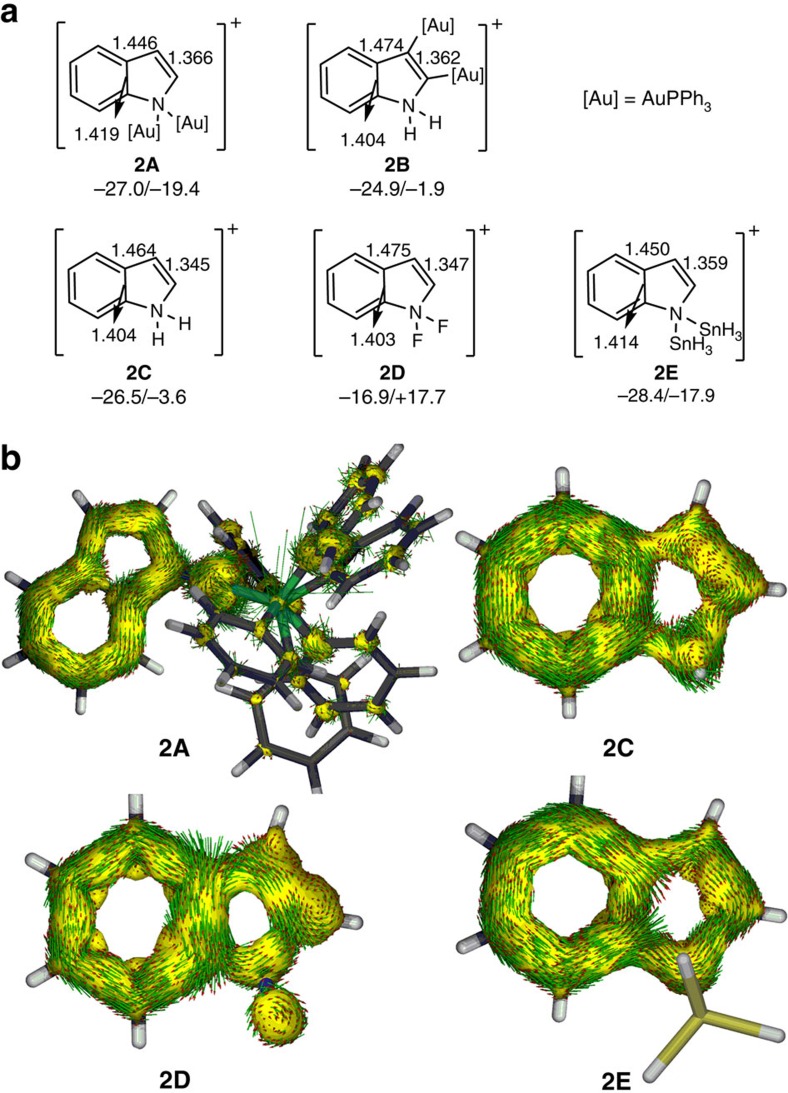
Hyperconjugative (anti)aromaticity in indolium derivatives. (**a**) The selected C-C bond lengths (Å), NICS(1)_zz_ values (p.p.m.) on the rings of **2A**-**2E**. The NICS(1)_zz_ values given before and after the ‘/' are those computed at 1 Å above the geometrical centres of six- and five-membered rings, respectively. (**b**) ACID isosurfaces of **2A**, **2C**, **2D** and **2E** by the *π* contribution. Current density vectors are plotted onto the ACID isosurface of 0.03 to indicate dia- and para-tropic ring currents. The magnetic field vector is orthogonal with respect to the ring plane and points upward.

The equalized bond lengths and good stability of the polyaurated heteroaryl complexes **2** and **10** prompt us to examine whether the auration has a special influence on the aromaticity of the heteroaryl moieties. Density functional theory (DFT) computations^42^ have then been carried out to help understand the aromaticity of the tetra- and octa-aurated heteroaryl complexes. For simplified model complexes **2'** and **10'**, the PH_3_ ligands was used to replace the PPh_3_ ligands of the cationic moiety of **2** and **10**. The nucleus-independent chemical shift (NICS) computations^43^,^44^ show that the NICS(1)_zz_ values^29^,^45^ for the five-membered ring (5MR) in **2'** and **10'** are –16.6 and –14.2 p.p.m., respectively (Supplementary Fig. 10). In general, negative values indicate aromaticity and positive values account for anti-aromaticity. These NICS(1)_zz_ values in the 5MR are in sharp contrast to that of the parent indolium **2C** (−3.6 p.p.m., Fig. 3), indicating an aromaticity switch from non-aromatic to aromatic. Note that the Au···Au distance in **2'** is 3.303 Å, which is much longer than the experimental value. Thus, the PPh_3_ ligands including dispersion corrections have been considered for the optimization of complexes **2A** and **2B**. Indeed, the Au···Au distance in **2A** becomes 2.889 Å, indicating that the large ligands and dispersion corrections play an important role in reproducing the aurophilic interaction. Further NICS calculations on complexes **2A** and **2B** suggest that it is the aurated substituents on the nitrogen atom rather than those on the *sp*^2^-hybridized carbon atom that are responsible for the aromaticity change. Specifically, the NICS(1)_zz_ value in the 5MR of **2A** is close to that of **2'**, whereas complexes **2B** has the similar NICS(1)_zz_ value with the parent indolium **2C**.

Previous study by Schleyer and Nyulászi^25^ indicated that distannylcyclopentadiene is nearly as aromatic as furan according to various aromaticity indices. To evaluate the magnitude of the aromaticity in **2A**, we performed the NICS calculation on distannylindolium **2E**. The NICS(1)_zz_ value in the 5MR of **2E** is –17.9 p.p.m., which is less negative than that of **2A**, suggesting higher aromaticity in **2A** over **2E**. Thus complex **2A** could be considered as the most aromatic indolium reported so far. The highest aromaticity of **2A** is also supported by highest delocalized C–C bonds in the 5MR. Specifically, the C–C bond length alternation (0.080 Å) is smallest among **2A**-**2E**. The important role of hyperconjugation in aromaticity of **2A** is further supported by the anti-aromaticity in the 5MR of difluoroindolium **2D**, indicated by the significant positive NICS(1)_zz_ value (+17.7−p.p.m.).

The hyperconjugatvie aromaticity in **2A** is further supported by the anisotropy of the current-induced density analysis^46^,^47^. As shown in Fig. 3b, the current density vectors plotted on the anisotropy of the current-induced density isosurface indicate a diatropic ring current in the 5MRs of **2A** and **2E** in the *π* system whereas the paratropic ring current is displayed in the 5MR of **2D**. No clear diatropic or paratropic ring current could be found in the 5MR of **2C**, suggesting its non-aromaticity. Thus, the concept of hyperconjugative aromaticity, firstly proposed by Mulliken^24^ and later confirmed by Schleyer, Nyulászi, O'Ferrall and Houk^25^,^26^,^27^,^28^, has now been first extended to transition metal substituted heterocycles^45^. More importantly, the aurated substituents can outperform the traditional main group substituents. These calculation results nicely confirm the aromatic essence of the geminally diaurated heteroaryls and account for the excellent stability of complexes **2** and **10**.

### Photoluminescent properties of aurated heteroaryl complexes

Previous studies have explored that the heavy-atom effect of gold significantly influences the spin–orbit coupling between states of different spin multiplicity, thus enhancing intersystem crossing from singlet to triplet excited states and facilitating radiative decay of the triplet excited state^48^,^49^. We therefore embarked on photophysical studies on four structurally well-defined polyaurated complexes **2**, **6**, **9** and **10**. **2** and **6** have similar absorption bands below 375 nm. In contrast, the absorption spectra of **9** and **10** extend to ∼475 nm (Supplementary Fig. 11). This bathochromic shift could be rationalized by the extended *π* system from **2** and **6** to **9** and **10**. In addition, the red shift is also reproduced qualitatively by time-dependent DFT calculations of **2'** and **10'** (the simplified complex of **10** with PH_3_ in place of PPh_3_). Specifically, the calculated intense absorption bands of complexes **2'** and **10'** at *λ*=321 and 382 nm are consistent with the bands observed at *λ*=307 and 355 nm of **2** and **10**, respectively. These computed absorption bands can be assigned to the electronic transitions HOMO→LUMO+1 and HOMO→LUMO+2 (HOMO is denoted as highest occupied molecular orbital whereas LUMO refers to lowest unoccupied molecular orbital, Supplementary Fig. 12), respectively, which are clearly a ligand-to-metal excitation nature.

**Figure 4:**
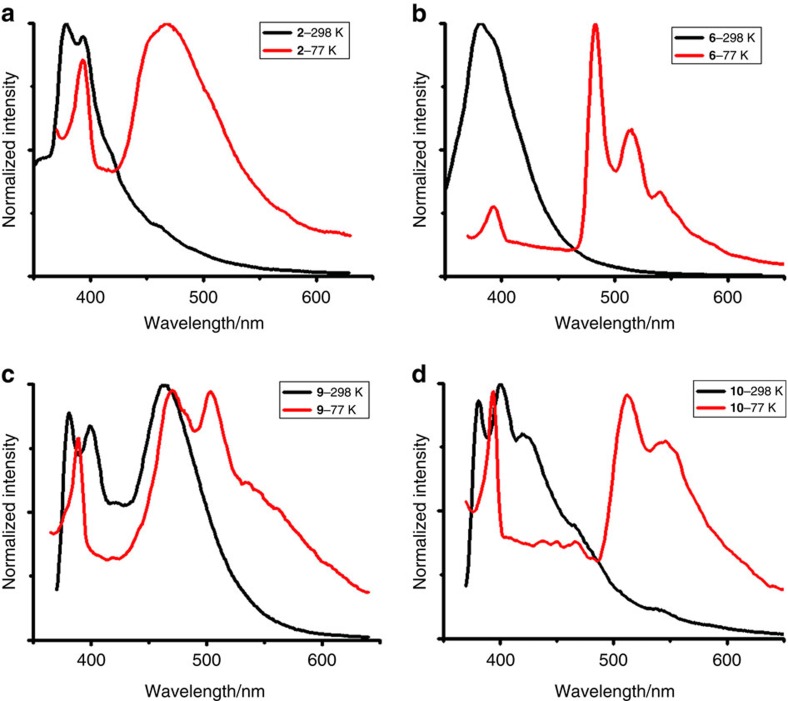
Emission spectra of polynuclear gold complexes. Emission spectra of (**a**) **2**, (**b**) **6**, (**c**) **9** and (**d**) **10** collected at 298 and 77 K in dichloromethane (*c*=5 × 10^−6^ M). Excitation: 325 nm (for **2** and **6**) and 345 nm (for **9** and **10**).

Upon excitation at 325 nm (for **2** and **6**) and 345 nm (for **9** and **10**) at 298 K in dichloromethane, all four complexes are luminous with the emission spectra spanning the range from 350 to 650 nm (Fig. 4). The luminescence intensity of all four complexes is diminished by exposure to oxygen (Supplementary Fig. 13). In addition, the emission spectra of **2**, **9** and **10** show vibronic structures with a mean peak-to-peak separation of 1,010, 1,190 and 1,247 cm^−1^, respectively. At low temperature (77 K), the emission from 450 to 550 nm becomes more intense and structured emission appears in the spectra of **6**, **9** and **10**. The emission lifetimes of the strongest emission for every complex at 77 K were collected and summarized in Table 1. The long lifetimes in microsecond-scale together with the oxygen-quenching of the emission and the large Stokes shifts indicate a phosphorescence parentage from a triplet-excited state. time-dependent DFT calculations on the model complex **2'** in the lowest triplet state (T_1_) indicate that the calculated emission of **2'** at *λ*_max_=383, 412 and 474 nm are consistent with the bands observed at *λ*=378, 393 and 464 nm of complex **2**, respectively. In addition, calculations reveal that the three emission bands are ligand-to-metal, metal-centred and ligand-centred, respectively (Supplementary Fig. 14). Gray and co-workers have once proposed a plausible excited-state hypothesis accounting for the temperature-dependent emission of aurated aryls^49^. It includes the decay of a singlet excited state to an excimer, which catalyses or pumps the formation of a triplet. High temperatures suppress excimer formation, resulting in emission difference. Furthermore, quantum yield measurements at 298 K revealed that the imido trinuclear gold(I) complexes **6** (37%) and **9** (14%) have higher yields than **2** (4%) and **10** (2%). This difference is possibly ascribed to the crowded arrangement of the AuPPh_3_ units in **6** and **9**, which impedes non-radiative decay processes by blocking molecular rotations and vibrations of PPh_3_ ligands.

## Discussion

The synthesis of the polyaurated heteroaryls **2** and **10** is unprecedented and unusual. Stepwise transformations from 2-ethynylaniline through **7**-to-**2** and from **8** through **9**-to-**10** highlight two alternative pathways towards the synthesis of polyaurated aryls no matter which step (the auration of the amine group and the *σ*-metalation of the TMS-substituted ethynyl group) takes place first. A common prerequisite for the synthesis of **2** and **10** is the *σ*-activation of the carbon–carbon triple bond, which accounts for the unsuccessful annulation of internal alkyne substrates such as **5**. Considering the isolobality of the proton and the LAu^+^ fragment, the μ_3_-imido aurated species is analogous to an ammonium species that is lack of nucleophilicity. We hypothesize that the NAu_3_ species in **3** and **9** exhibit nucleophilicity on condition that the trinuclear gold cluster aggregate dissociates into low nuclearity species. This speculation is partially evidenced by the transformation of **9** to **10**. In this transformation process, the stoichiometric ratio of **9** to Ph_3_PAuCl is 1:2. The number of [AuPPh_3_] units in **9** plus two equivalents Ph_3_PAuCl is equal to that of **10**, while the number of the nitrogen-bonded gold unit decreases from three in **9** to two in **10**. This result suggests the release of a AuPPh_3_ unit from the imido NAu_3_ aggregate. This AuPPh_3_ unit may translocate to finally attach on a carbon centre of the resulting heteroaryls. If replacing Ph_3_PAuCl (2 equiv.) by the same amount of Cy_3_PAuCl (Cy=cyclohexyl) in the **9**-to-**10** transformation, the ESI-MS of the reaction mixture revealed several Au_8_-benzodipyrrole species containing different ligand combinations such as [6PPh_3_+2PCy_3_], [5PPh_3_+3PCy_3_] and [4PPh_3_+4PCy_3_], suggesting the dissociation and intermolecular exchange of phosphine ligands (Supplementary Fig. 15).

On the other hand, hyperconjugation, a weak interaction in chemistry, has a strong effect on the aromaticity. Simply tuning the substituents can lead to an aromaticity switch from non-aromaticity to (anti)aromaticity. Previous studies on hyperconjugative aromaticity mainly focus on the carbocycles. Examples on heterocycles are particularly rare and all the substituents reported so far contain main group elements only. Our calculations first demonstrate that the substituent containing the transition metal gold is also able to work for hyperconjugative aromaticity. More importantly, the introduction of *d*-orbitals from the transition metal can have a stronger effect on the hyperconjugative aromaticity in comparison with that of *p*-orbitals from traditional main group elements, thus opening an avenue to the design of various novel metalla-aromatics.

In summary, we have demonstrated a facile synthetic method of producing polyaurated heteroaryls. Two alternative pathways by activating the ethynyl group at first and then metalating the amine group or *vice versa* both accomplished the synthesis of unprecedented polyaurated indole and benzodipyrrole complexes. Theoretical analysis revealed an unexpected aromaticity change due to the hyperconjugation caused by the introduction of aurated substituents, which can perform better than the traditional main group substituents, extending the concept of hyperconjugative aromaticity to the transition metal-involved heterocycles. This study showcases a promising approach to acquire more complicated and intricate organometallics by forming saturated polymetalated units. Structure and reactivity comprehension of these polymetalated organometallics foresees deep research in bonding nature and mechanistic studies and stimulates the advancement of more efficient and versatile synthetic methods.

## Methods

### General

All commercially available reagents were used as received. Flash column chromatography was performed on silica gel (100–200). The solvents used in this study were dried by 4 Å molecular sieves. ^1^H-NMR, ^13^C-NMR and ^31^P-NMR spectra were recorded by using 400 MHz spectrometers. Chemical shifts are reported in ppm versus tetramethylsilane with either tetramethylsilane or the residual solvent resonance used as an internal standard. Synthetic procedures for complexes **6**, **7** and **9** are summarized in Supplementary Methods. ESI-MS spectra of complexes **6** and **9** are shown in Supplementary Figs 16 and 17, respectively. ^1^H-, ^31^P- and ^13^C-NMR spectra of complexes **7** (Supplementary Figs 18–20), **6** (Supplementary Figs 21–23), **2** (Supplementary Figs 24–26), **9** (Supplementary Figs 27–29) and **10** (Supplementary Figs 30–32) are shown in Supplementary Information.

### Synthesis of 2

Complex **7** (10 mg, 0.017 mmol) and oxotris((triphenylphosphine)gold) tetrafluoroborate (25.7 mg, 0.017 mmol) were dissolved in CHCl_3_ (2 ml) with stirring at room temperature over 20 min. Addition of hexane into the reaction mixture afforded a white solid of **2**. Yield: 82% (30 mg, 0.014 mmol). Single crystals of **2** were obtained by vapour diffusion of diethyl ether into a CHCl_3_ solution of **2**. ^1^H-NMR (400 MHz, CDCl_3_): δ 7.87–7.80 (m, 2H); 7.48 (dd, *J*=12.3, 7.8 Hz, 10H); 7.38 (dd, *J*=12.4, 7.5 Hz, 20H); 7.30 (d, *J*=7.7 Hz, 12H); 7.15–7.11 (m, 2H); and 7.05 (td, *J*=7.8, 1.8 Hz, 18H). ^13^C-NMR (100 MHz, CDCl_3_): δ 134.2, 134.1, 132.1, 131.6, 130.5, 129.9, 129.5, 129.4, 129.3, 129.2, 128.6, 122.7, 121.9, 120.1 and 114.7. ^31^P-NMR (162 MHz, CDCl_3_): δ 39.43, 33.61. IR (KBr, cm^−1^): 3052, 1964, 1890, 1812, 1667, 1615, 1587, 745, 710 and 692; HR-MS (ESI): calcd. for [M-BF_4_]^+^ (C_80_H_64_Au_4_NP_4_) 1950.2646, found 1950.2641. Elemental Analysis: Calcd. for (C_80_H_64_Au_4_BF_4_NP_4_+1/2(CHCl_3_)): C, 46.85; H, 3.37; and N, 0.65. Found: C, 46.88; H, 3.31; and N, 0.78.

### Synthesis of 10

To a mixture of **9** (32.3 mg, 0.010 mmol) and 2 equiv. of PPh_3_AuCl (9.9 mg, 0.020 mmol) in dichloromethane (2 ml) were added a methanolic solution (0.5 ml) of potassium fluoride (2.3 mg, 0.030 mmol) dropwise and 20 equiv. of K_2_CO_3_ (27.6 mg, 0.20 mmol). The mixture was stirred overnight in the dark. The solvent was removed by vacuum and the residue was redissolved in CH_2_Cl_2_. The resulting mixture was filtered through celite. Addition of Et_2_O to the filtrate afforded a yellow solid of **10**. Yield: 71% (28.4 mg, 0.0071, mmol). Single crystals of **10** were obtained by vapour diffusion of diethyl ether into a CHCl_3_ solution of **10** after 2 days. ^1^H-NMR (400 MHz, CDCl_3_): δ 8.29 (s, 2H); 7.48-7.36 (m, 64H); and 7.09-6.97 (m, 56H). ^13^C-NMR (100 MHz, CDCl_3_): δ 134.1, 131.9, 131.4, 130.6, 130.0, 129.2 and 105.8. ^31^P-NMR (162 MHz, CDCl_3_): δ 39.48, 33.56. IR (KBr, cm^−1^): 3049, 1964, 1890, 1812, 1585, 746, 709 and 692; HR-MS (ESI): calcd. for [M–2BF_4_]^2+^ (C_154_H_122_Au_8_N_2_P_8_) 1911.7428; found 1911.7428. Elemental analysis: Calcd. for (C_154_H_122_Au_8_B_2_F_8_N_2_P_8_+CH_2_Cl_2_): C, 45.98; H, 3.22; and N, 0.68. Found: C, 45.67; H, 3.12; and N, 0.85.

### X-ray crystallographic analysis

Data for complexes **2**, **6**, **9** and **10** (CCDC-1435045) were collected at 173 K with Mo-Kα radiation (*λ*=0.71073 Å) on a Rigaku Saturn 724+ CCD diffractometer with frames of oscillation range 0.5°. All structures were solved by direct methods, and non-hydrogen atoms were located from difference Fourier maps. All non-hydrogen atoms were subjected to anisotropic refinement by full-matrix least-squares on *F*^2^ by using the SHELXTL program unless otherwise noticed^50^. All crystal structure figures were drawn by using X-seed program^51^. Structural refinement details are summarized in Supplementary Methods. See also Supplementary Data 1.

### Photophysical measurements

Photophysical measurement of low-temperature glass samples was carried out with the solid sample loaded in a quartz tube inside a quartz-walled Dewar flask. Liquid nitrogen was placed into the Dewar flask for low temperature (77 K). Luminescence quantum yields for solution samples were measured by the optical dilute method. All values are relative to quinine sulfate in 1.0 M H_2_SO_4_ as being 0.55 as the reference. Luminescent decay experiments were measured on an Edinburgh FLS920 spectrometer with μF920 microsecond flash lamp (pulse width<2 μs) as light source.

### Data availability

The authors declare that the data supporting the findings of this study are available within the article and its Supplementary Information files. Experimental details for **6**, **7** and **9**, characterization of compounds, Cartesian coordinates and energies of all the structures appearing in Fig. 3, and computational details can be found in Supplementary Figs 1–32 and Supplementary Methods.

## 

## Additional information

**Accession codes**: The X-ray crystallographic coordinates for structures reported in this article have been deposited at the Cambridge Crystallographic Data Centre (CCDC), under deposition number CCDC-1435042 (**2**), 1435041 (**6**), 1435040 (**9**) and 1435045 (**10**). These data can be obtained free of charge from the Cambridge Crystallographic Data Centre via www.ccdc.cam.ac.uk/data_request/cif.

**How to cite this article:** Yuan, J. *et al*. Synthesis of tetra- and octa-aurated heteroaryl complexes toward probing aromatic indoliums. *Nat. Commun.* 7:11489 doi: 10.1038/ncomms11489 (2016).

## Supplementary Material

Supplementary Information Supplementary Figures 1-32, Supplementary Methods and Supplementary References

Supplementary Data 1 Combined crystallographic information files for complexes **2**, **6**, **9** and **10**.

## Figures and Tables

**Table 1:** Photophysical parameters.

**Complex**	**Emission**		
	**T/K**	***λ***_**max**_**/nm (*****τ***_**o**_**/μs)**	**Φ**_em_*
**2**	298	378, 393, 464	0.04
	77	393, 468(219)†	
**6**	298	381	0.37
	77	393, 482(609)†, 515, 540	
**9**	298	380, 398, 421, 462	0.14
	77	389, 470(62)^‡^, 505, 567	
**10**	298	381, 400, 421, 468, 541	0.02
	77	394, 512(53)‡, 546	

^*^Measured at 298 K using quinine sulfate dihydrate as a standard.

^†^Measured in a 2-methyltetrahydrofuran glass at 77 K.

^‡^Measured in a EtOH-MeOH-CH_2_Cl_2_ (40:20:1 v/v) glass at 77 K.
