# Supplementary material for: Synthesis of tetra- and octa-aurated heteroaryl complexes towards probing aromatic indoliums
Source: Nat Commun. 2016 May 17;7:11489. doi: 10.1038/ncomms11489 (PMC4873667; doi:10.1038/ncomms11489)
Supplement: Supplementary Information — Supplementary Figures 1-32, Supplementary Methods and Supplementary References [file ncomms11489-s1.pdf]

## Supplementary Figures

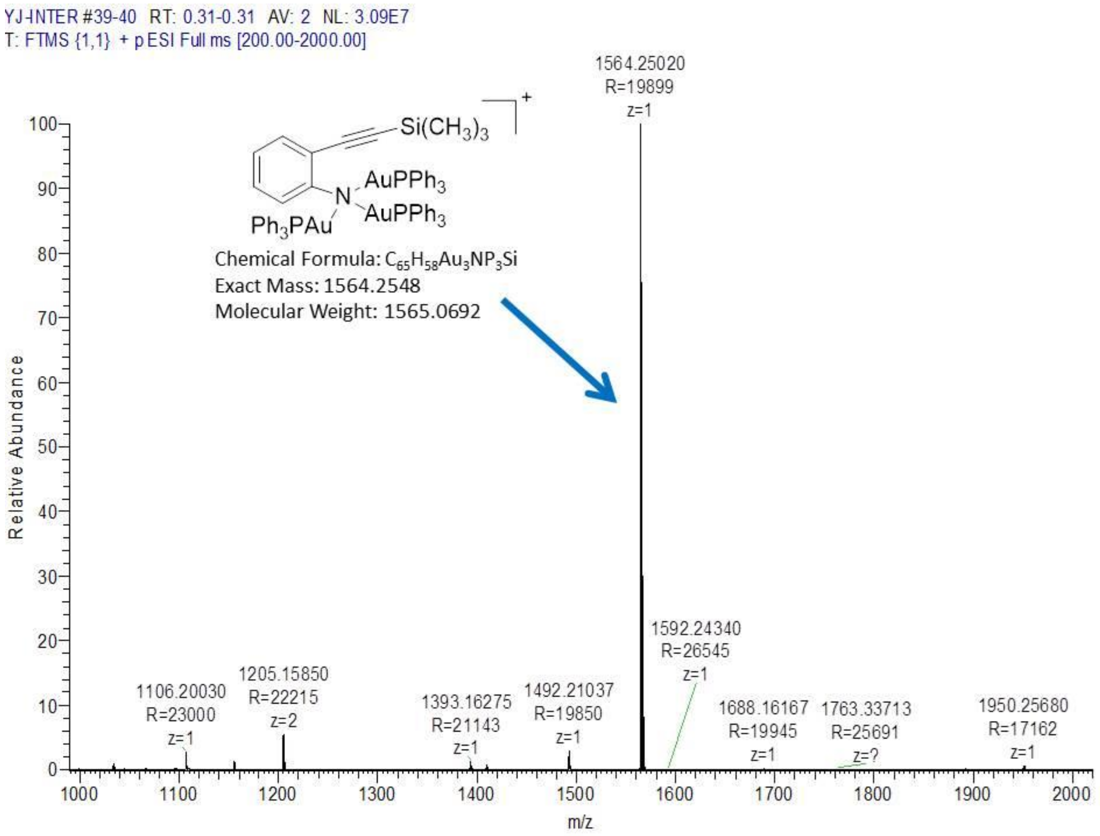


**Supplementary Figure 1.** ESI-MS spectrum of the reaction mixture of **1** and $[O(AuPPh_3)_3](BF_4)$, indicating the intermediate role of **3**.

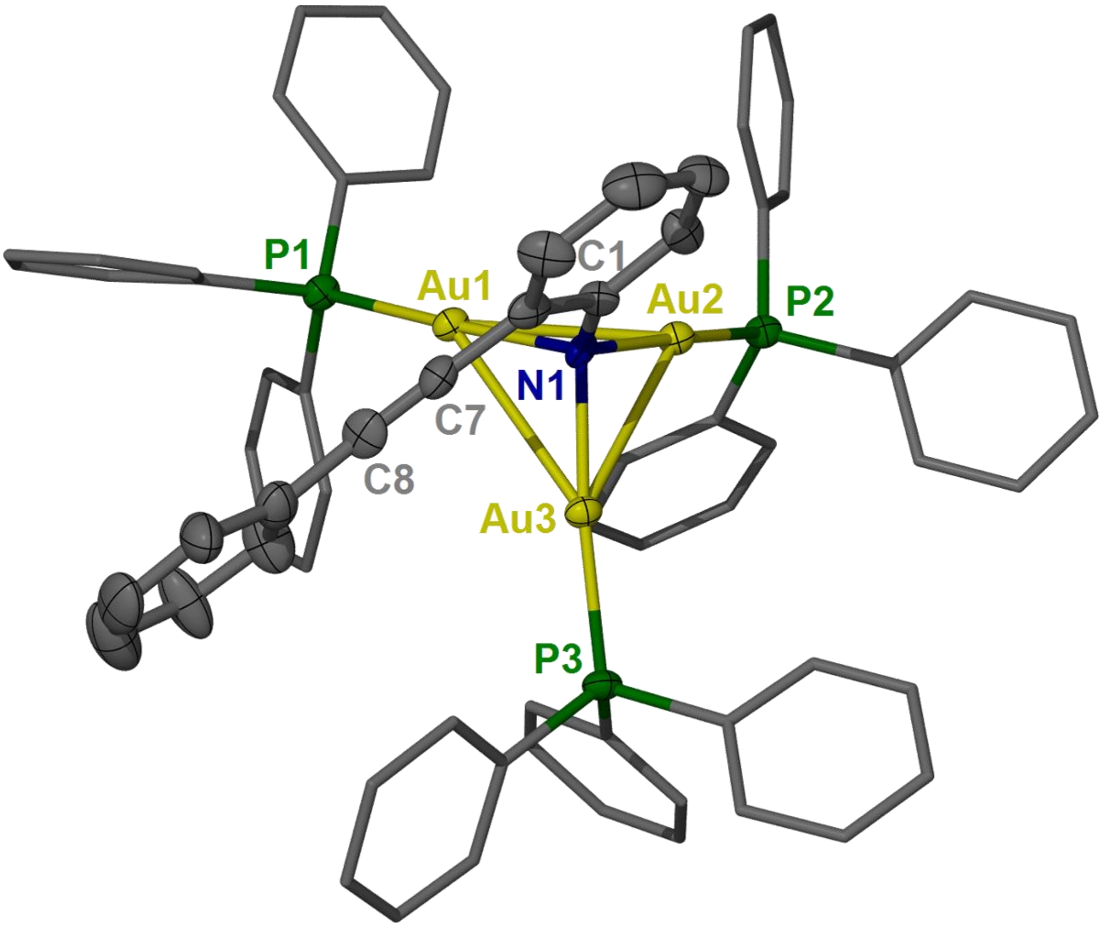


**Supplementary Figure 2.** X-ray crystal structure of complex **6**. Only an unsymmetrical $NAu_3$ unit is shown here. $BF_4$ and hydrogen atoms are omitted for clarity. Selected bond lengths and distances (Å): Au1-N1 2.052(7); Au2-N1 2.064(7); Au3-N1 2.081(7); C7-C8 1.177(15); Au1-P1 2.240(3); Au2-P2 2.236(3); Au3-P3 2.238(3); Au1 ···Au2 3.001(1); Au1 ···Au3 3.176(1); Au2 ···Au3 3.038(1).

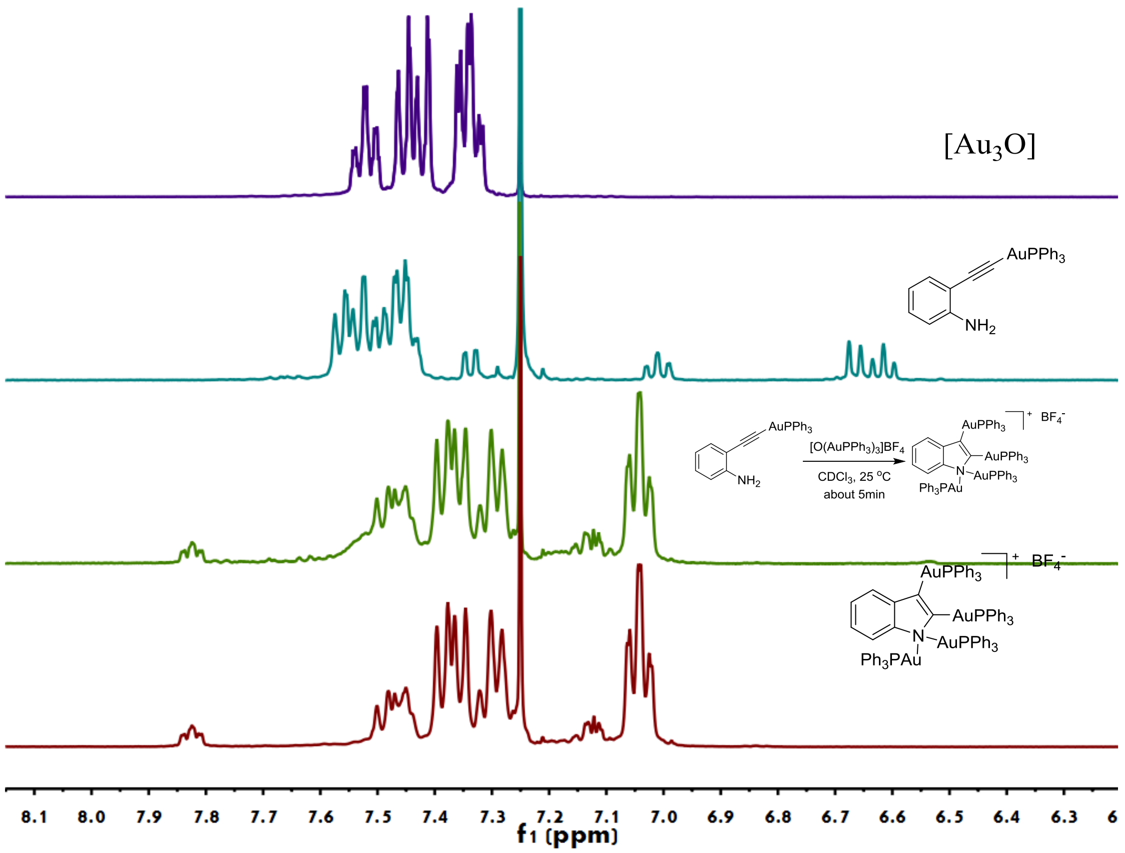


**Supplementary Figure 3.** $^{1}$H-NMR spectrum (the third from top) of the reaction mixture of **7** and [O(AuPPh$_3$)$_3$](BF$_4$) (1 equiv.), suggesting a rapid and quantative transformation to produce **2** within five minutes.

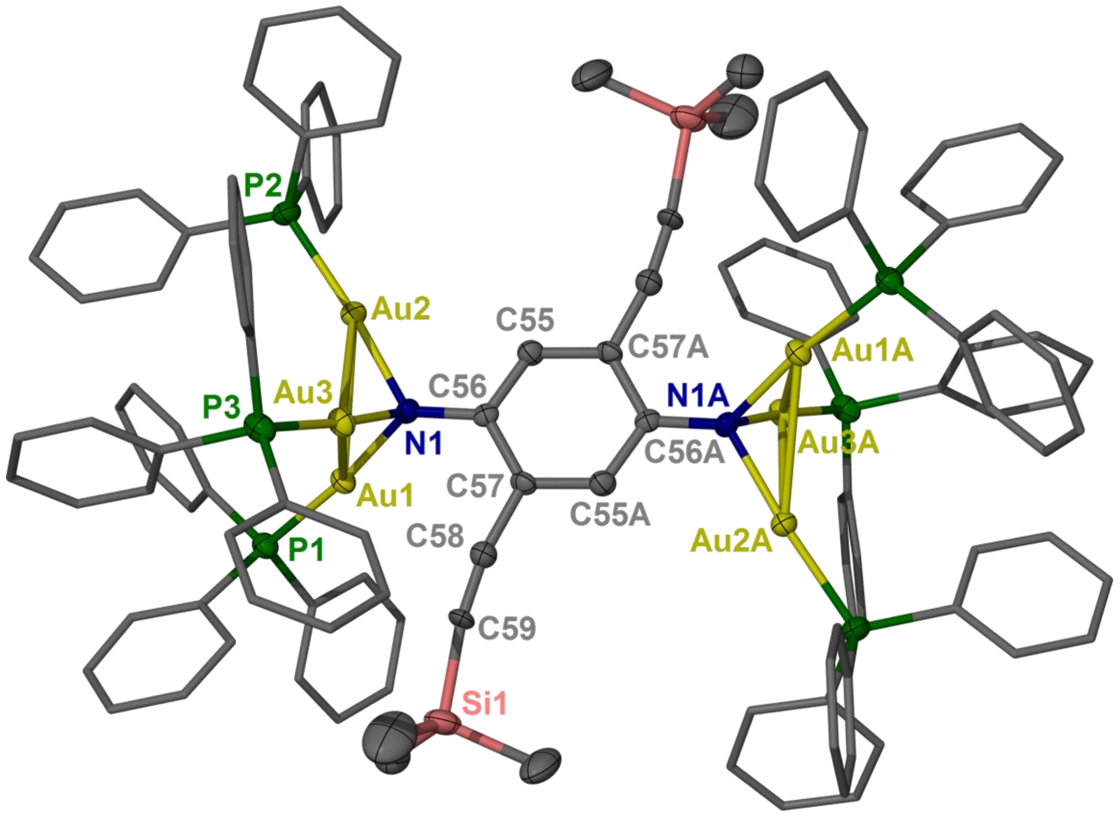


**Supplementary Figure 4.** X-ray crystal structure of complex **9**. $BF_4$ and hydrogen atoms are omitted for clarity. Selected bond lengths and distances (Å): Au1-N1 2.055(7); Au2-N1 2.065(6); Au3-N1 2.081(7); C58-C59 1.205(12); Au1-P1 2.240(2); Au2-P2 2.240(2); Au3-P3 2.237(2); Au1 ···Au2 3.073(1); Au1 ···Au3 3.050(1); Au2 ···Au3 2.982(1).

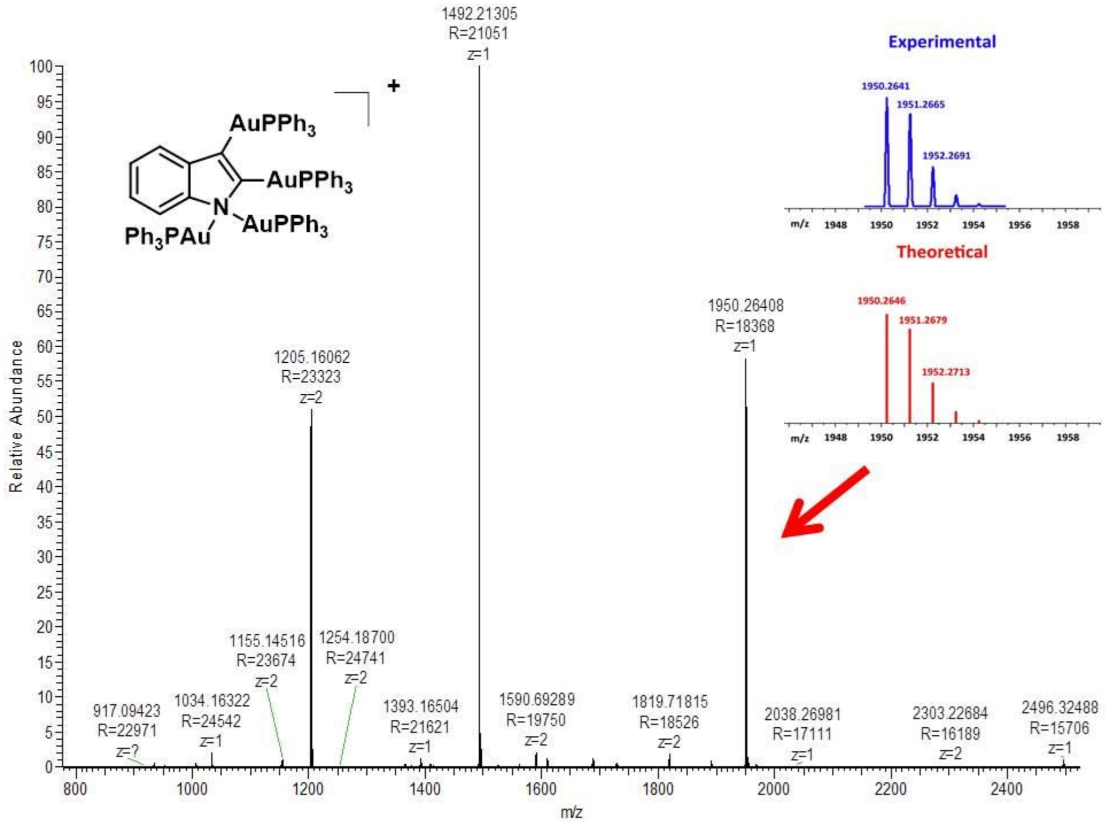


**Supplementary Figure 5.** ESI-MS spectrum of the chloroform solution sample of complex **2**.

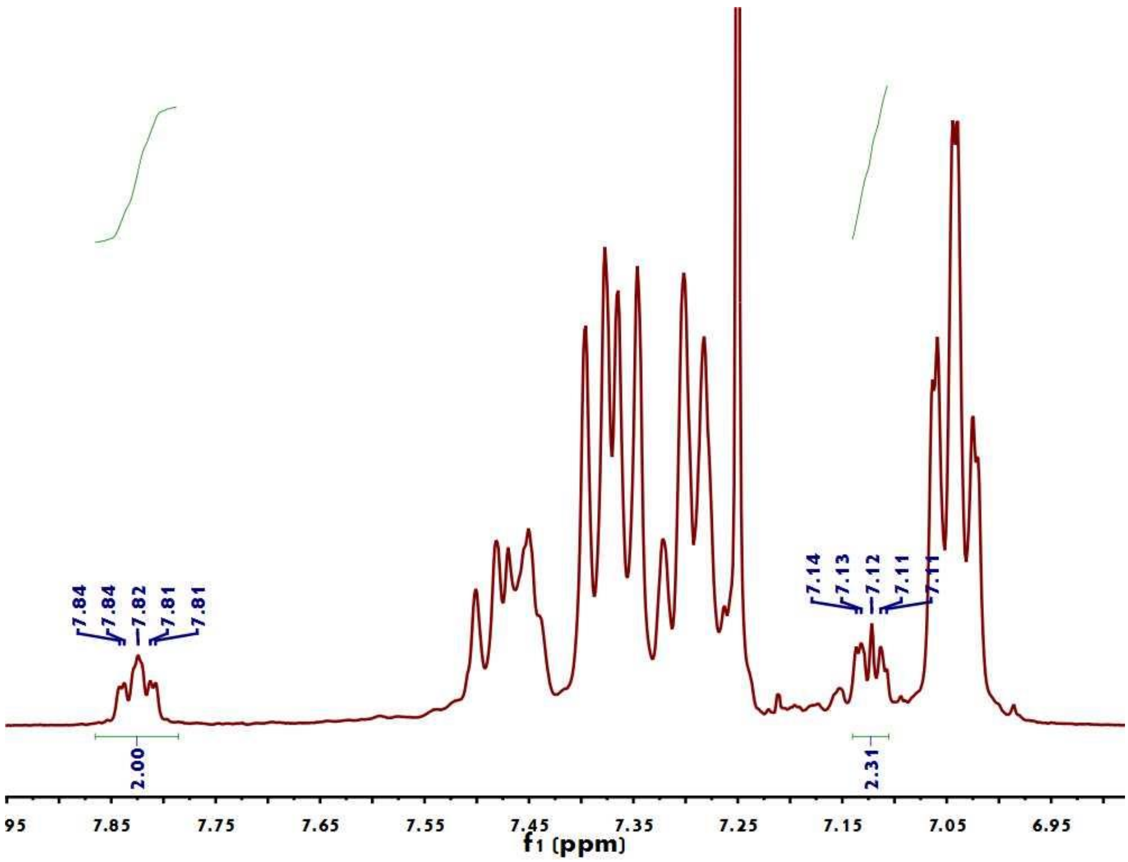


**Supplementary Figure 6.** $^{1}$H-NMR spectrum ($CDCl_3$, 400 MHz) of **2** clearly showed two multiplets at 7.12 and 7.82 ppm, corresponding to the four protons on the benzene ring of the central indolyl skeleton.

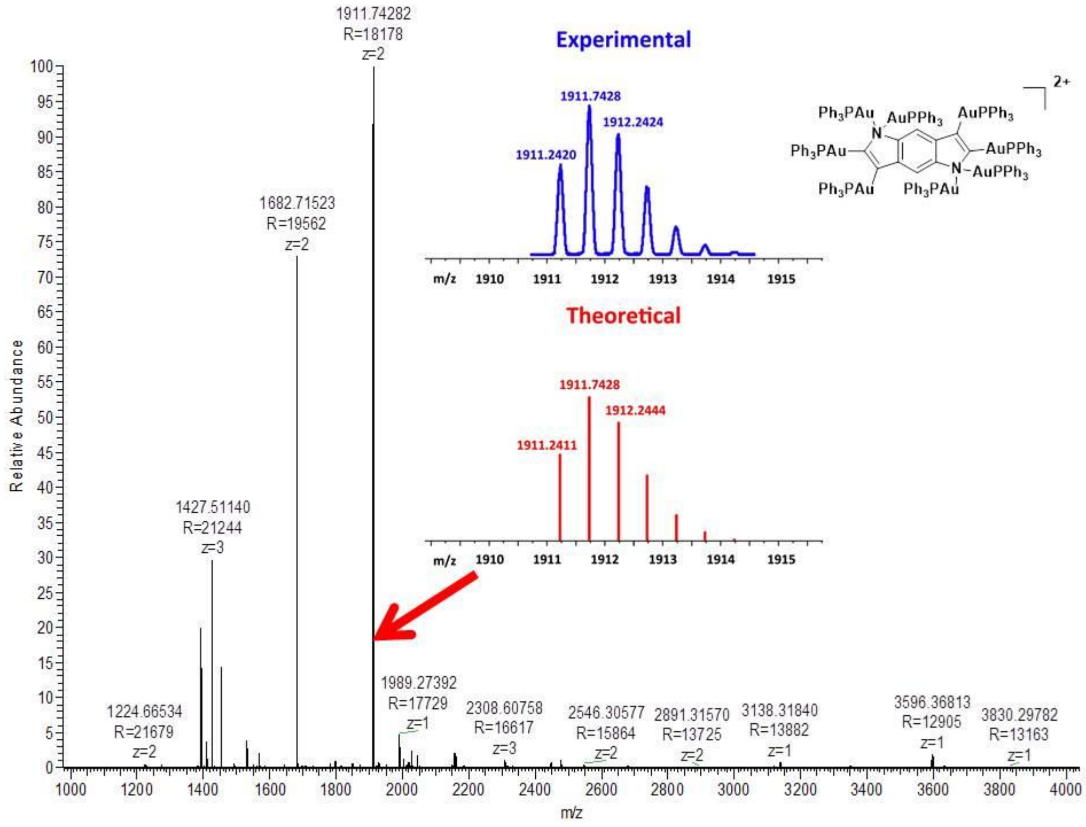


**Supplementary Figure 7.** ESI-MS spectrum of the chloroform solution sample of complex **10**.

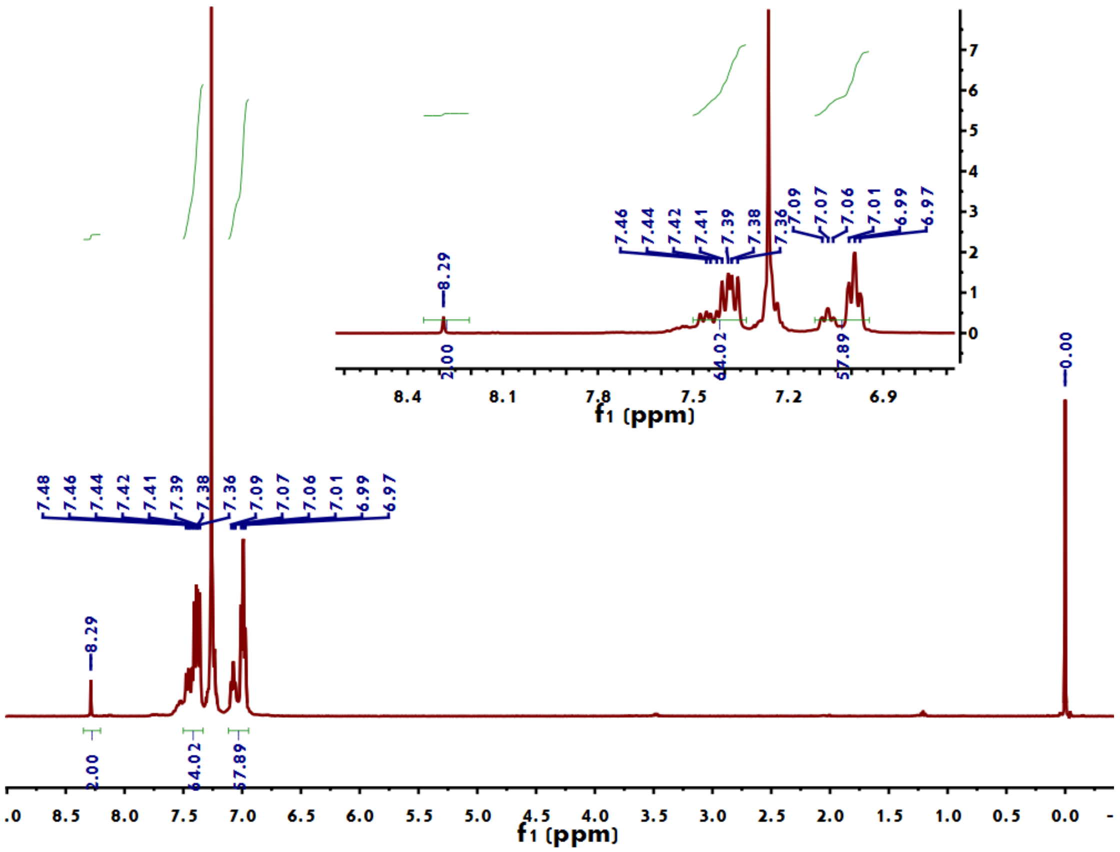


**Supplementary Figure 8.** $^{1}$H-NMR spectrum ($CDCl_3$, 400 MHz) of **10**.

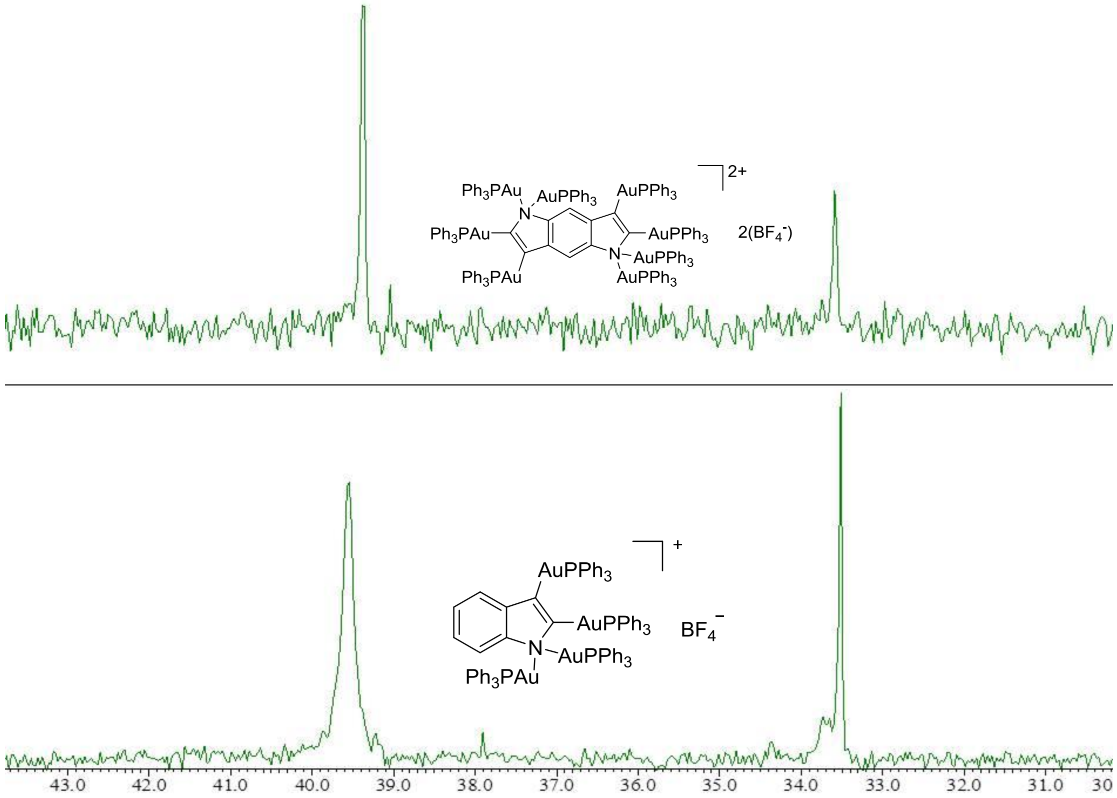


**Supplementary Figure 9.** $^{31}$P-NMR spectrum ($CDCl_3$, 162 MHz) of **2** and **10**.

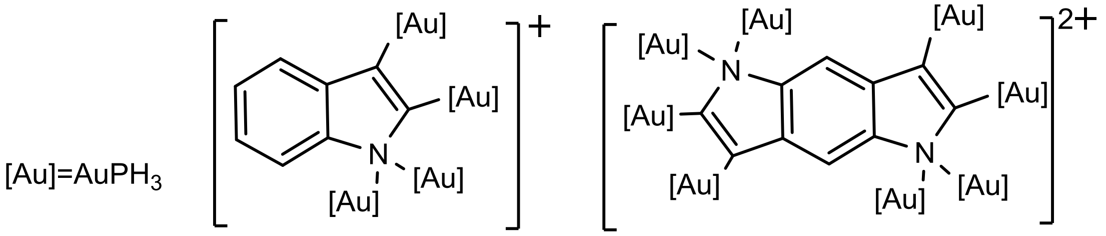


**2'** -26.1/-16.6

**10'** -24.0/-14.2

**Supplementary Figure 10.** The NICS(1)$_{zz}$ values (ppm) on the rings of **2'** and **10'** computed at the TPSS/6-31G(d) level. The NICS(1)$_{zz}$ values given before and after the '/' are those computed at 1 Å above the geometrical centres of six- and five-membered rings, respectively.

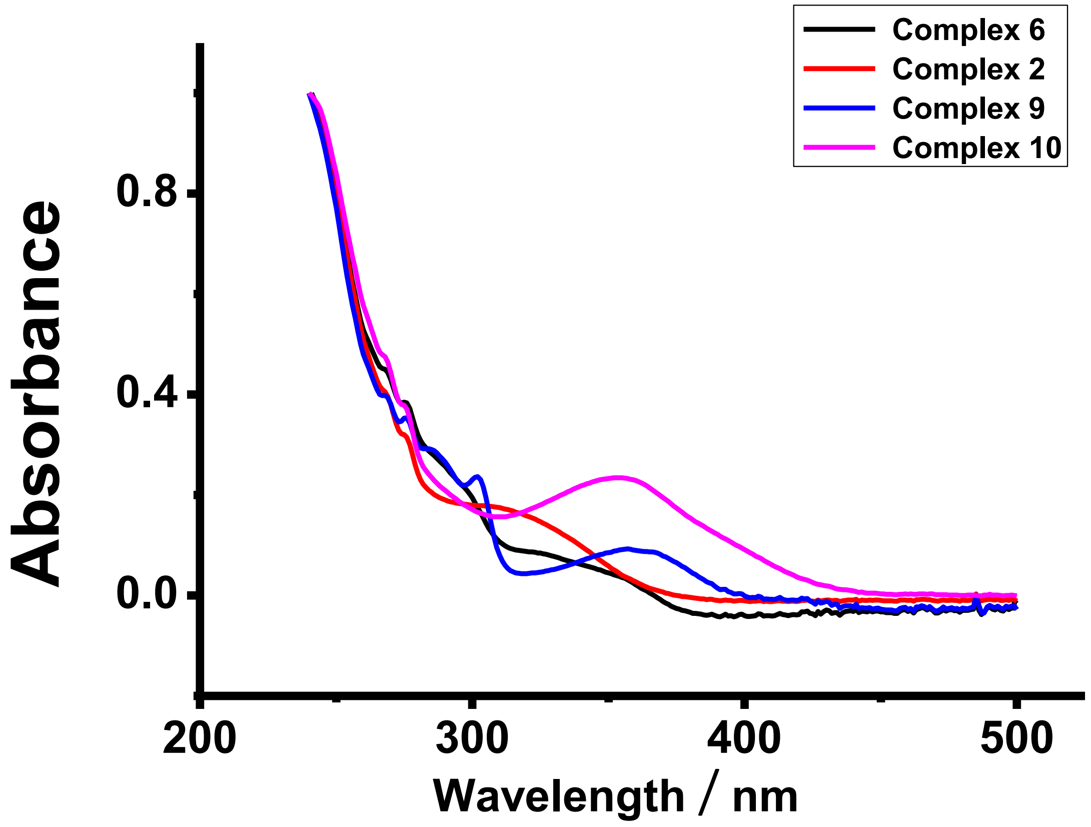


**Supplementary Figure 11.** UV-vis absorption spectra of complexes **2, 6, 9** and **10**.

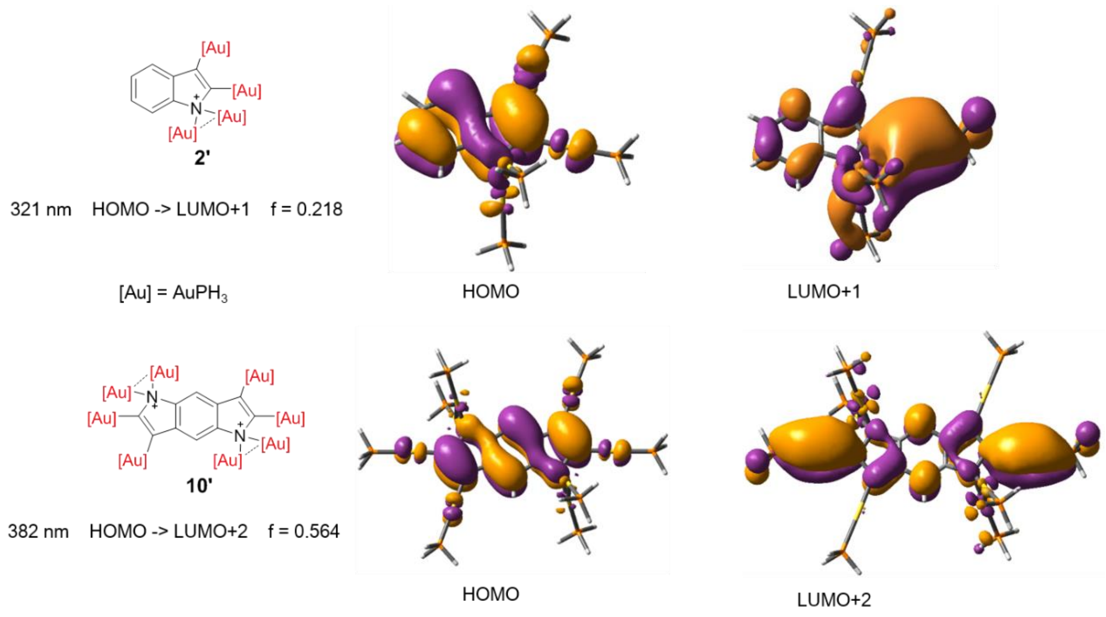


**Supplementary Figure 12.** Calculated intense absorption bands, oscillator strengths f, and electronic transitions of model complexes **2'** and **10'**.

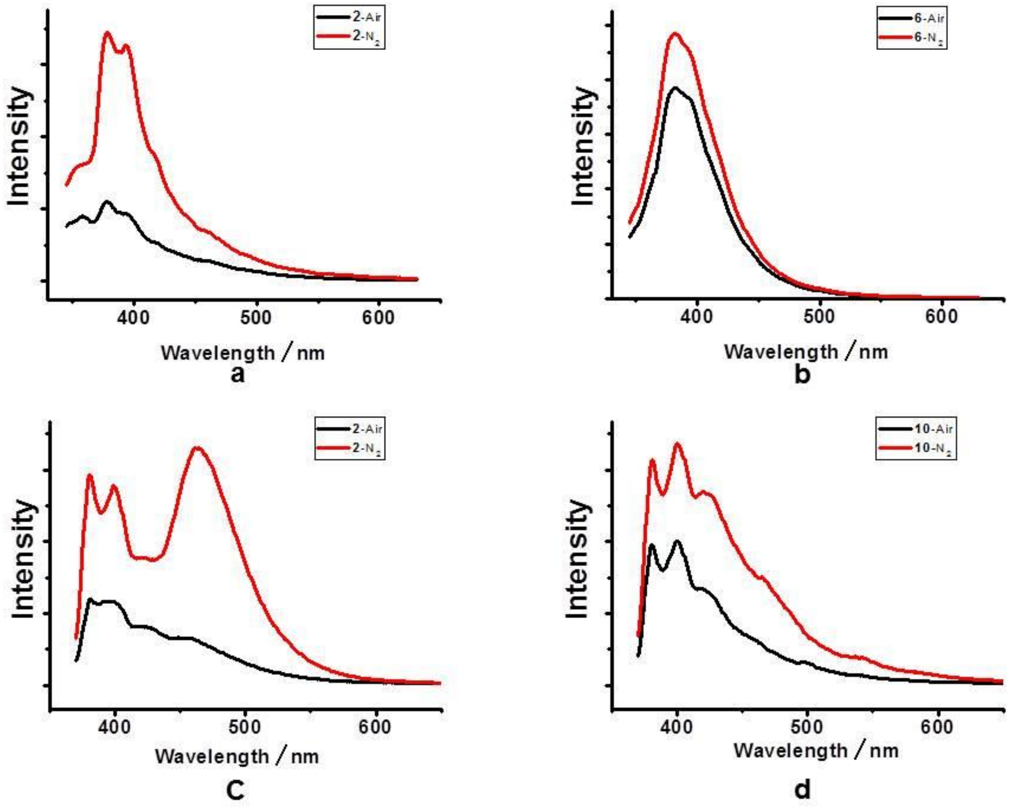


**Supplementary Figure 13.** Emission spectra (298 K) of (a) **2**, (b) **6**, (c) **9** and (d) **10** in degassed dichloromethane (red line) and after exposure to oxygen (black line). Excitation: 325 nm (for **2** and **6**) and 345 nm (for **9** and **10**).

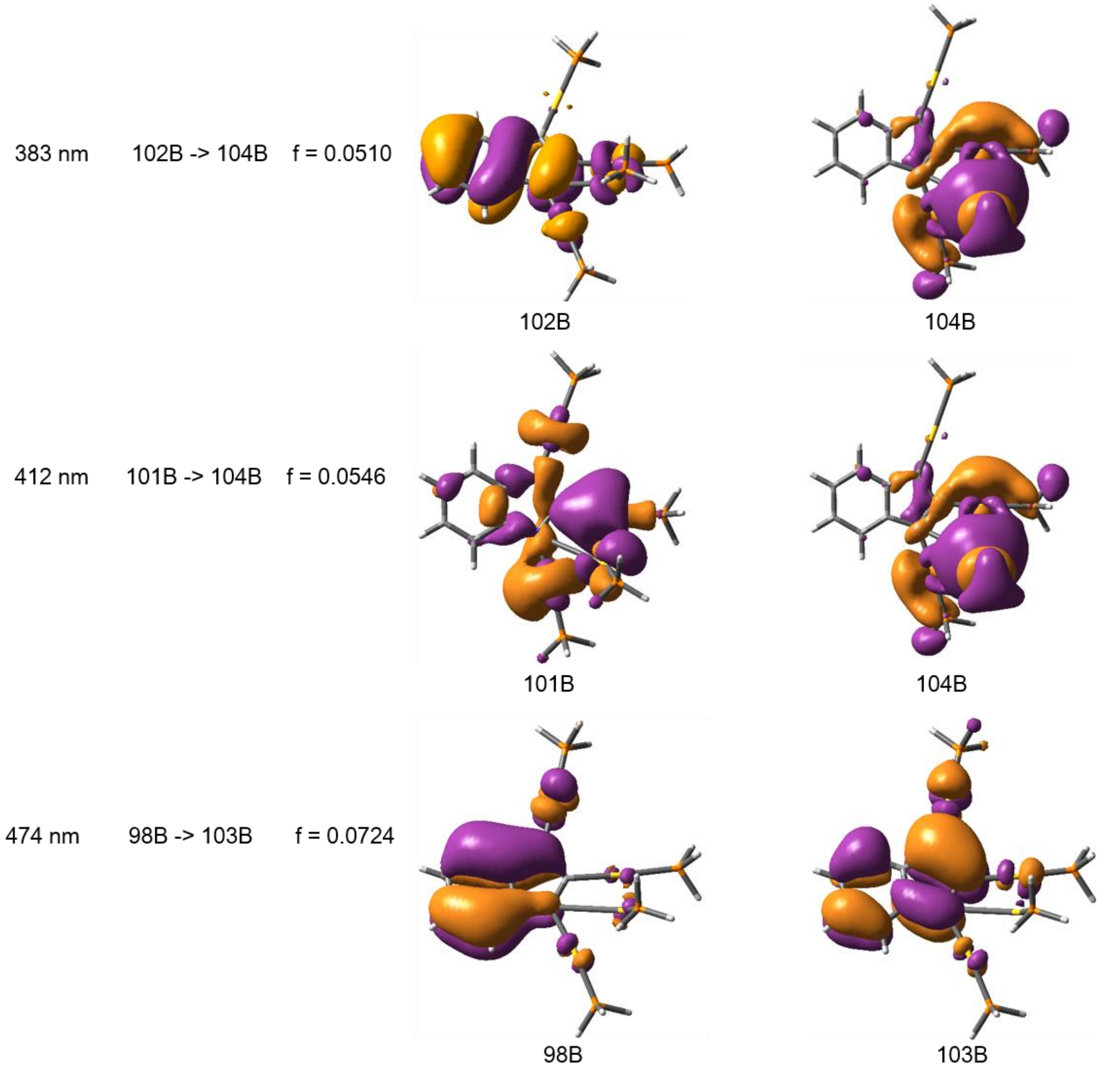


**Supplementary Figure 14.** Calculated excitation wavelengths, oscillator strengths f, and electronic transitions of the lowest triplet states of model complex **2'**. B denotes a beta orbital.

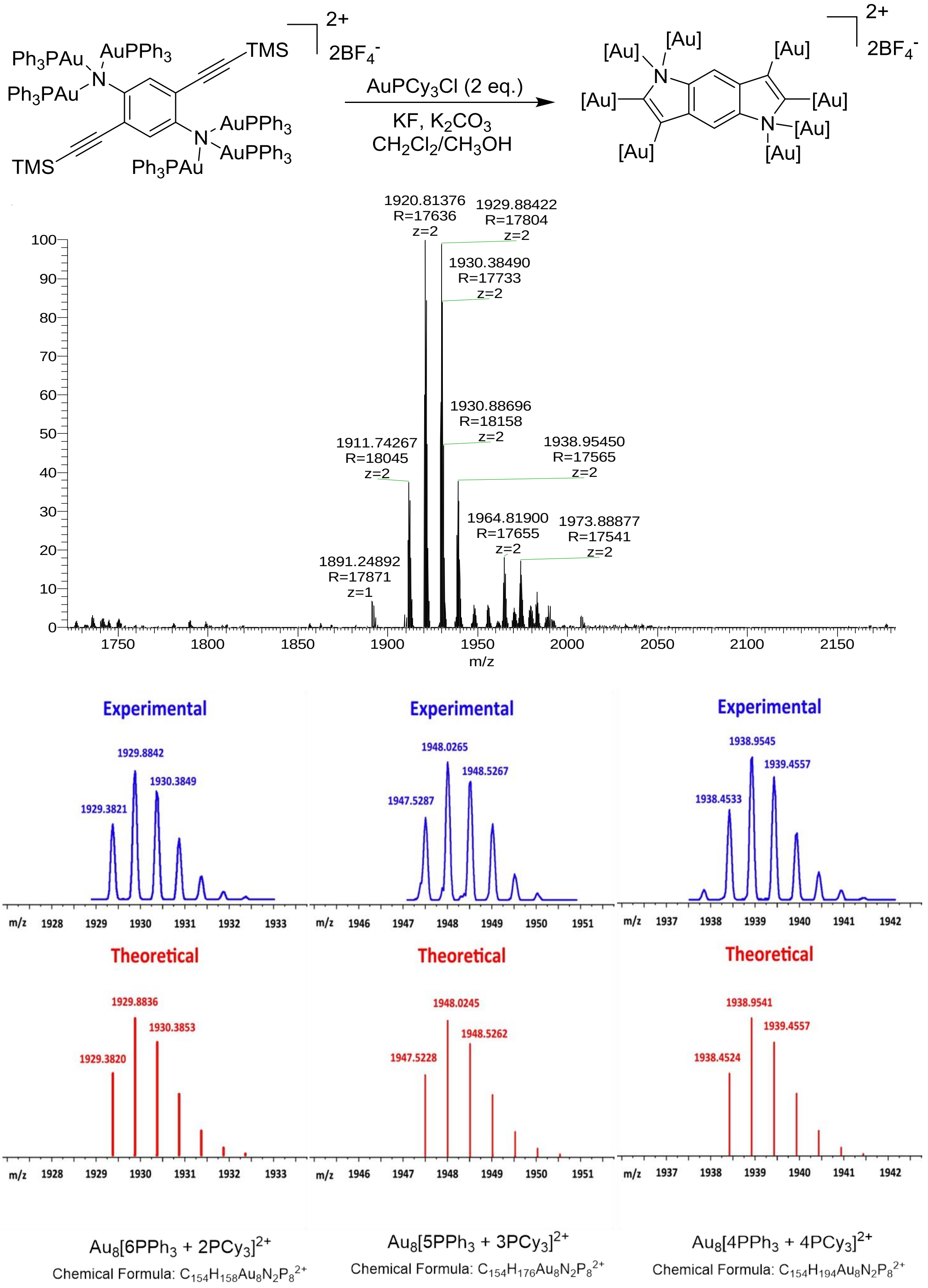


**Supplementary Figure 15.** ESI-MS spectrum of the reaction of **9** with $Cy_3PAuCl$, yielding several $Au_8$-benzodipyrrole species containing different ligand combinations.

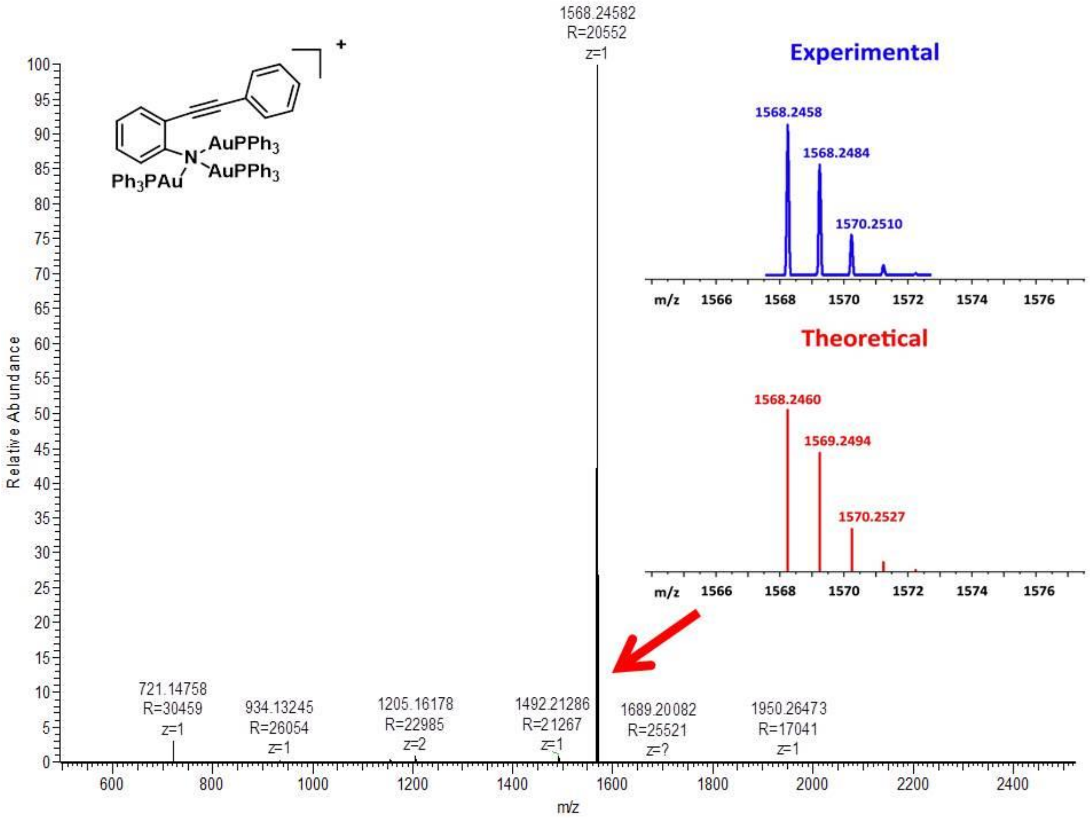


**Supplementary Figure 16.** ESI-MS spectrum of the chloroform solution sample of complex **6**.

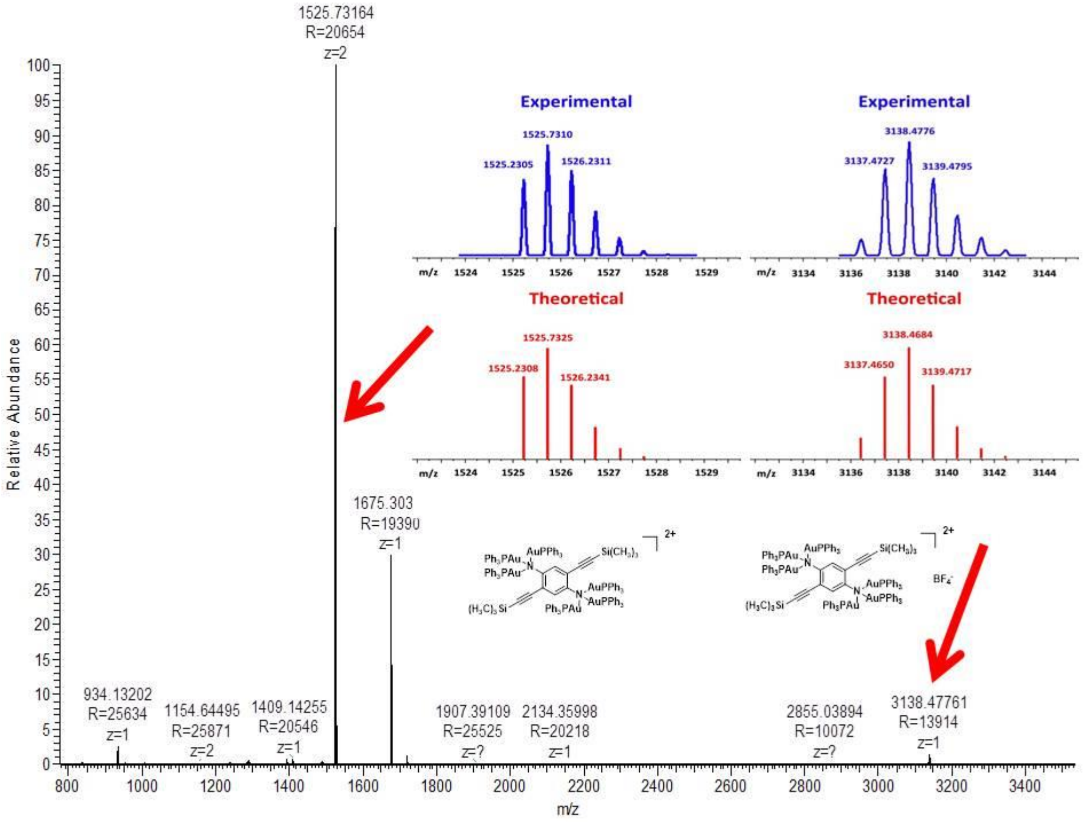


**Supplementary Figure 17.** ESI-MS spectrum of the chloroform solution sample of complex **9**.

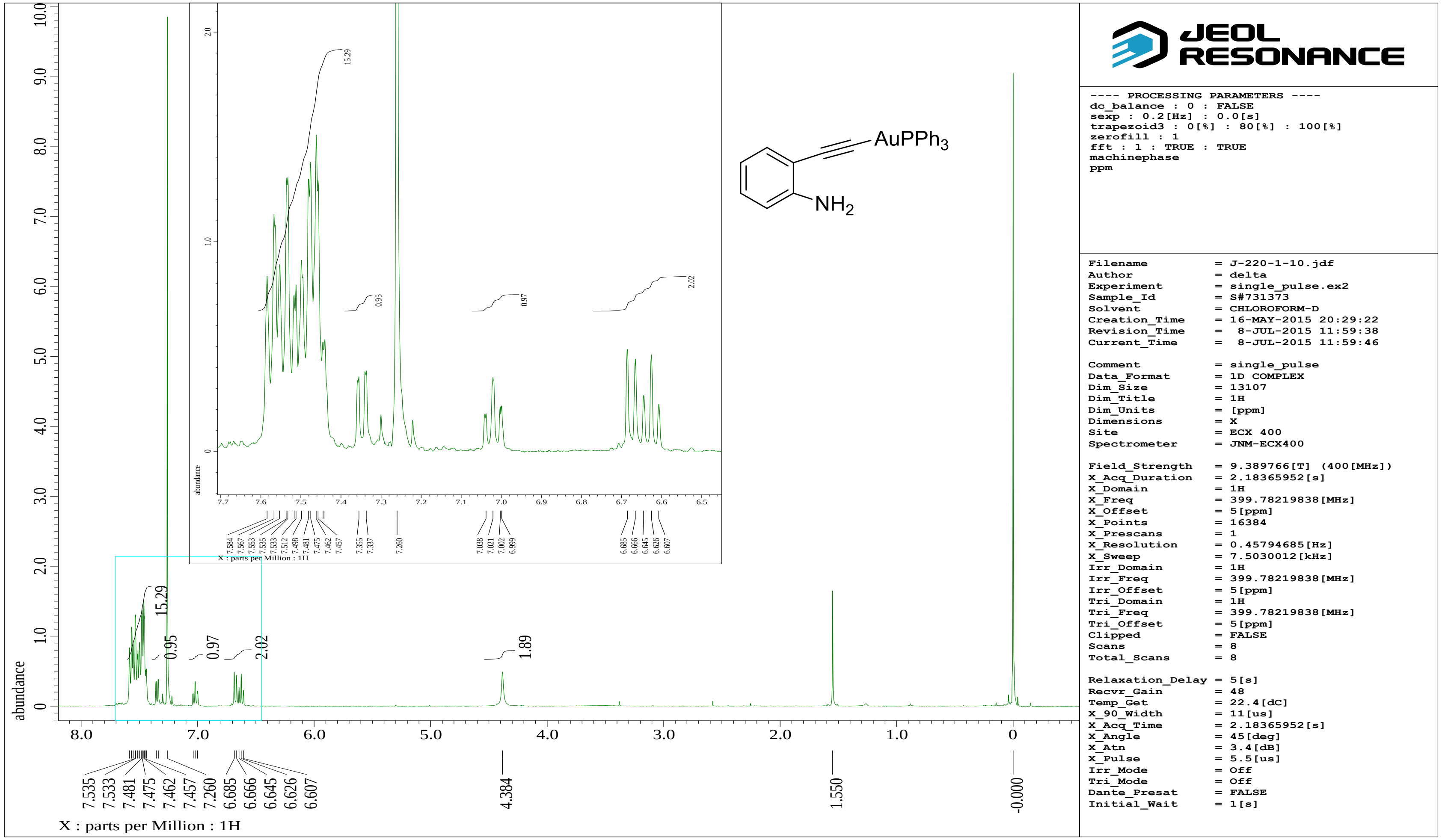


**Supplementary Figure 18.** $^1$H-NMR spectra of **7** in $CDCl_3$ at 298K.

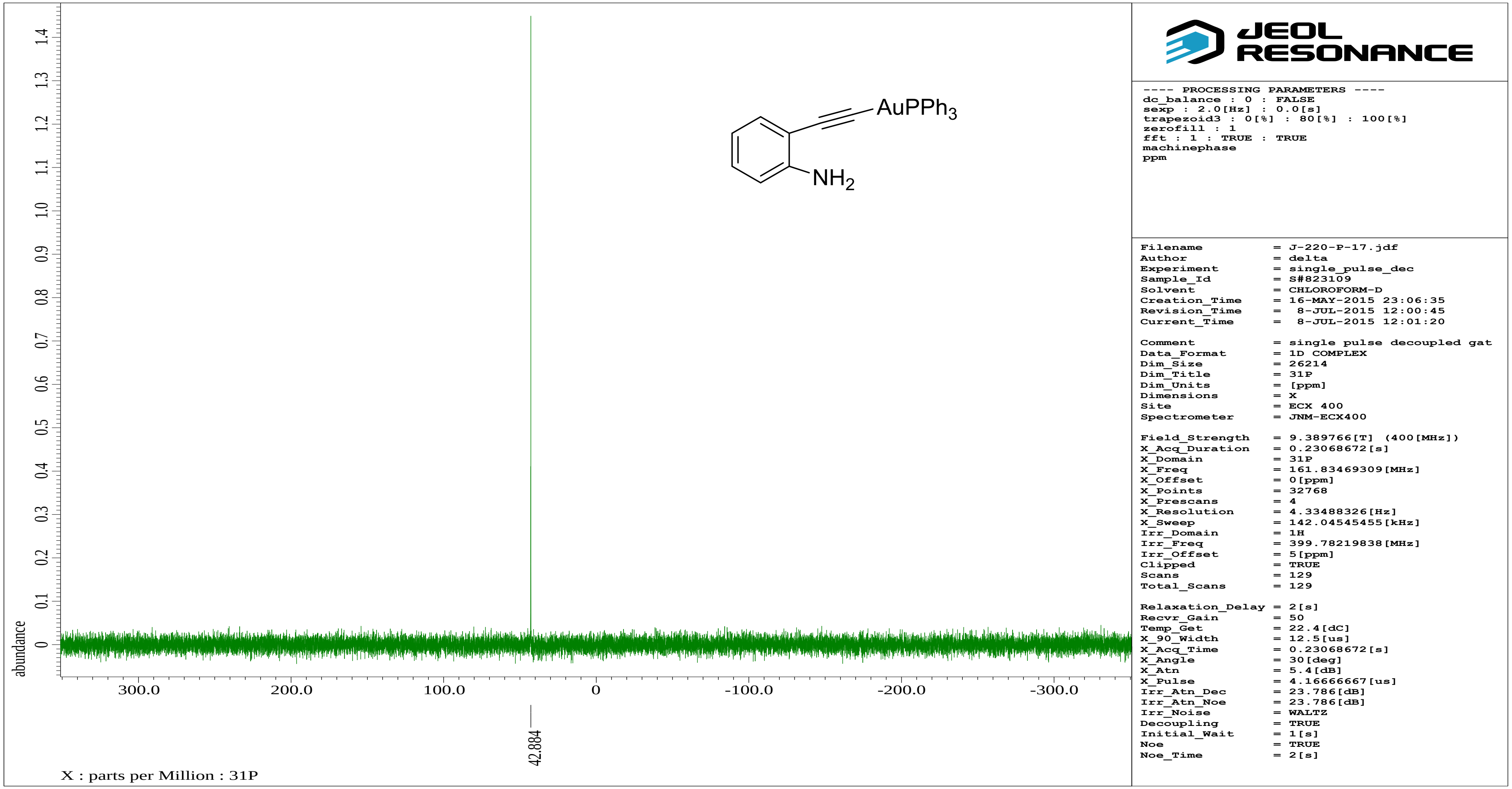


**Supplementary Figure 19.** $^{31}$P-NMR spectra of **7** in $CDCl_3$ at 298K.

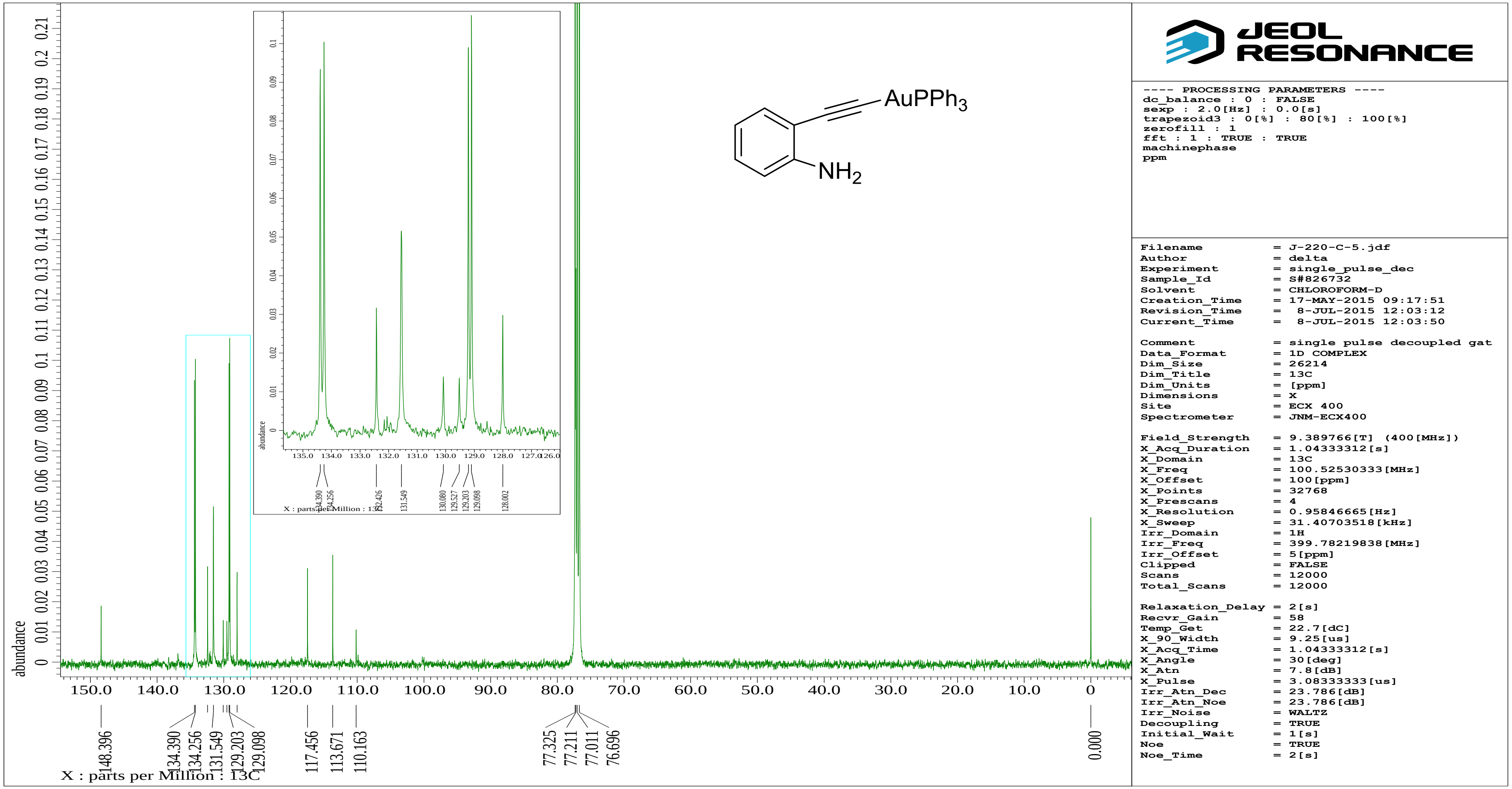


**Supplementary Figure 20.** $^{13}$C-NMR spectra of **7** in $CDCl_3$ at 298K.

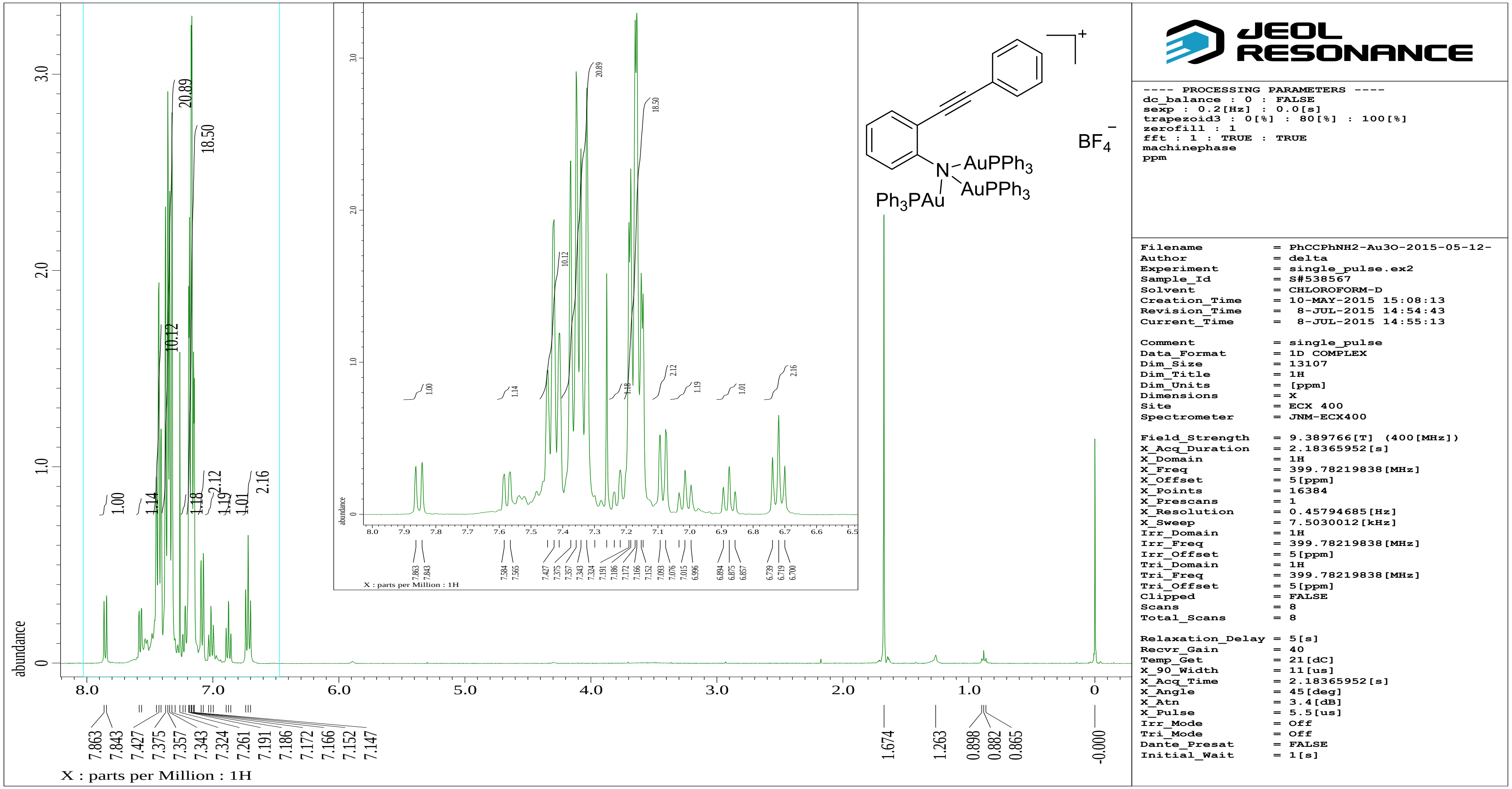


**Supplementary Figure 21.** $^{1}$H-NMR spectra of **6** in $CDCl_3$ at 298K.

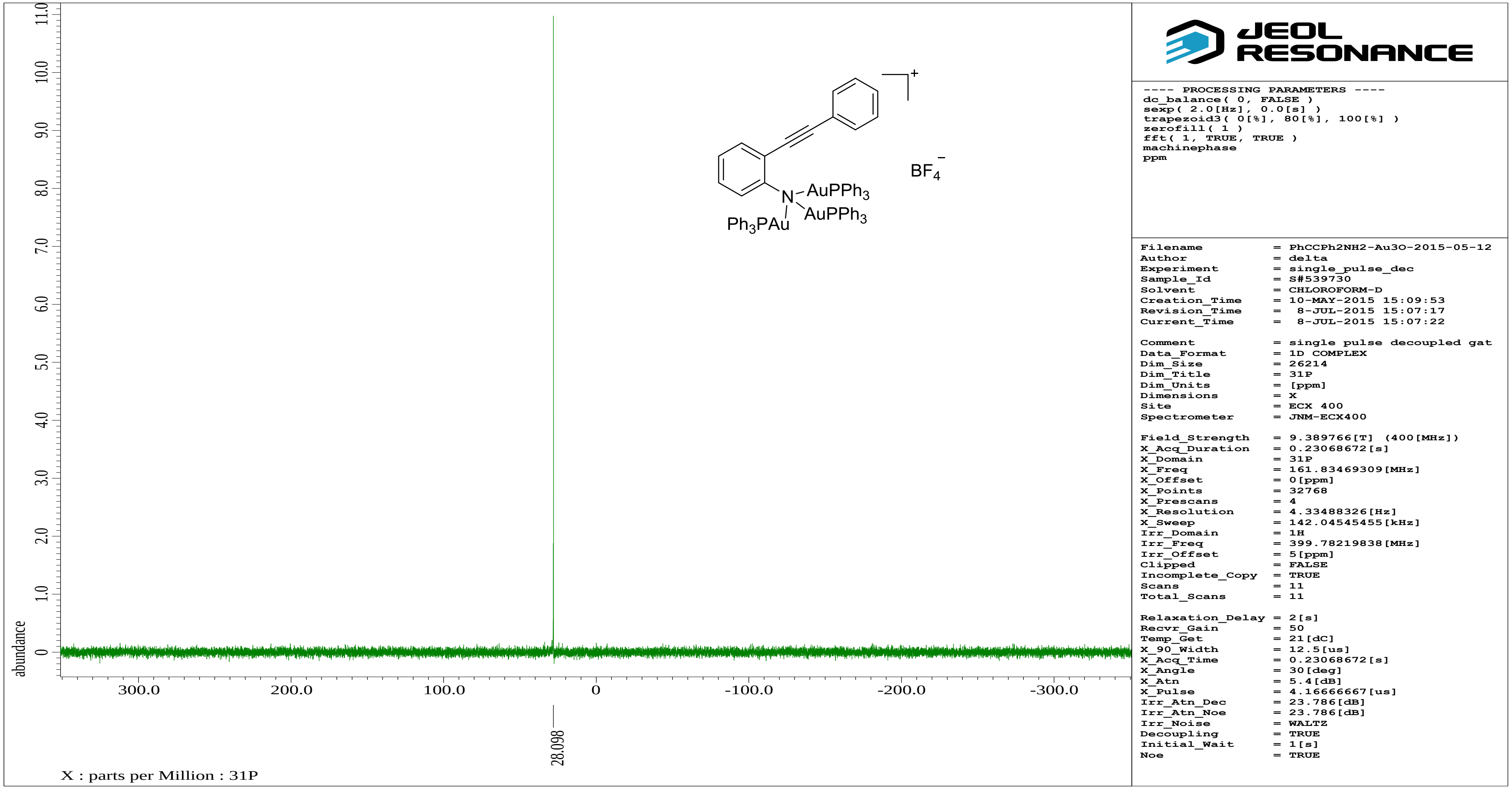


**Supplementary Figure 22.** $^{31}$P -NMR spectra of **6** in $CDCl_3$ at 298K.

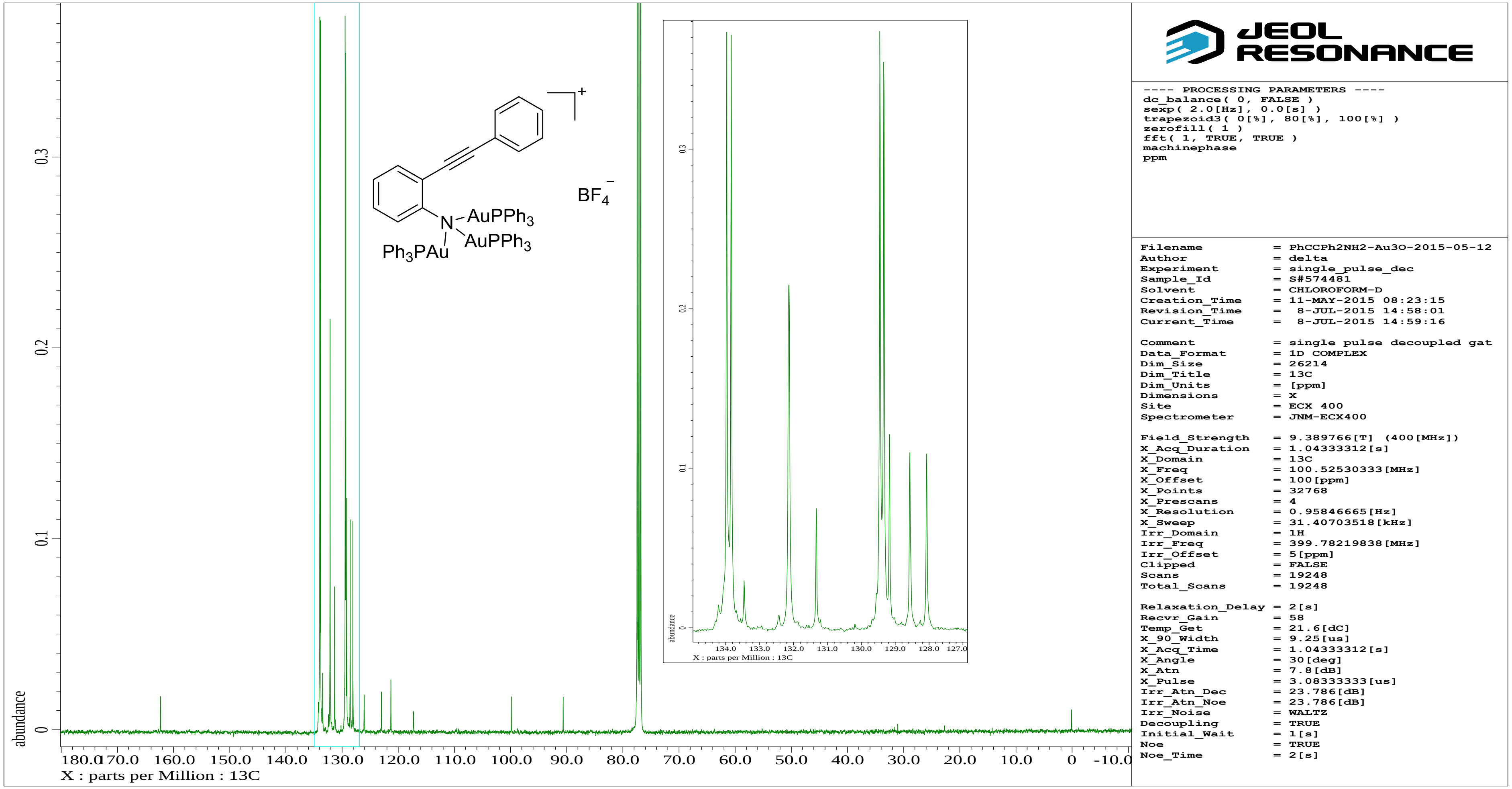


**Supplementary Figure 23.** $^{13}$C-NMR spectra of **6** in $CDCl_3$ at 298K.

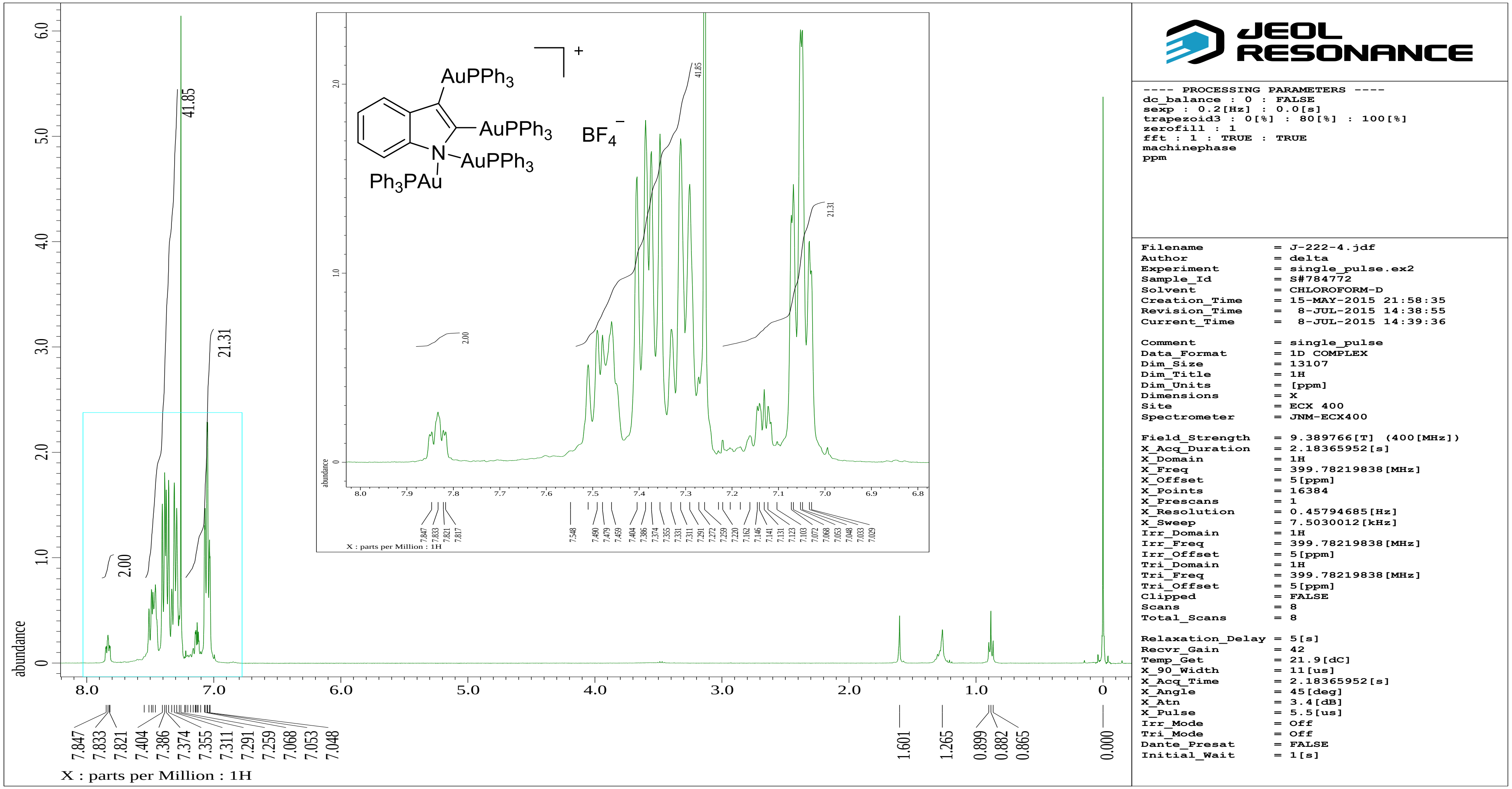


**Supplementary Figure 24.** $^1$H-NMR spectra of **2** in $CDCl_3$ at 298K.

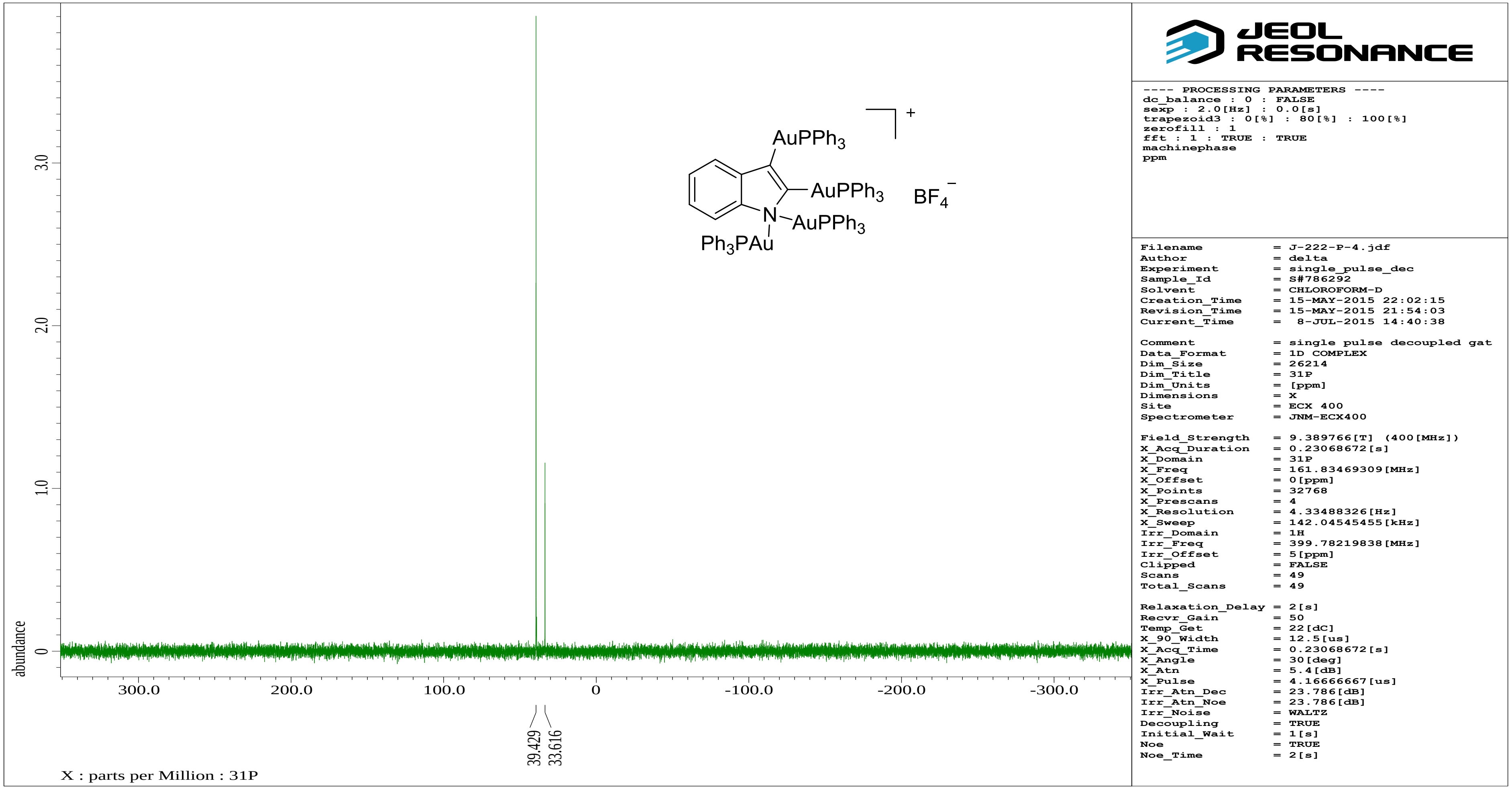


**Supplementary Figure 25.** $^{31}$P-NMR spectra of **2** in $CDCl_3$ at 298K.

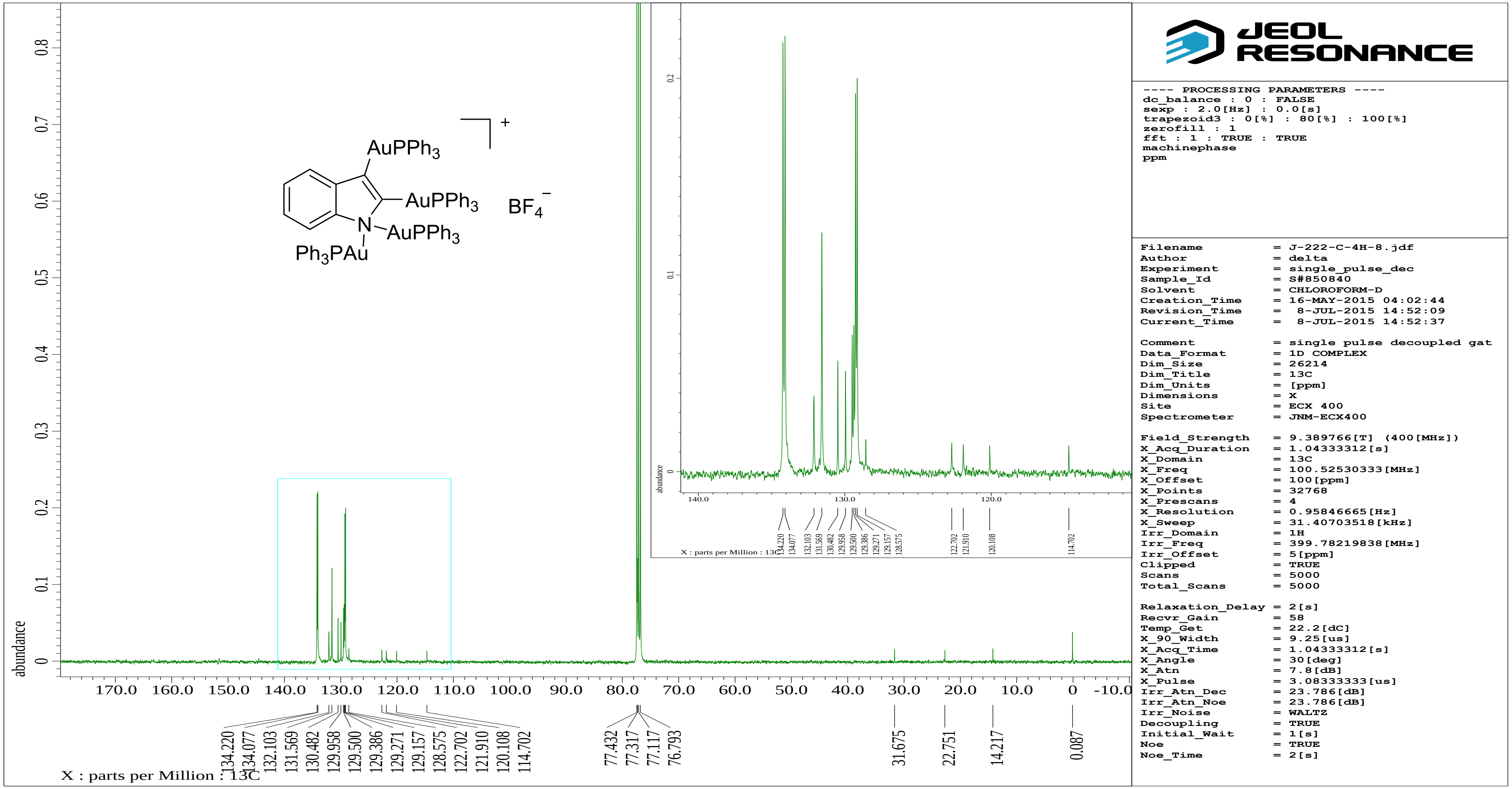


**Supplementary Figure 26.** $^{13}$C-NMR spectra of **2** in $CDCl_3$ at 298K.

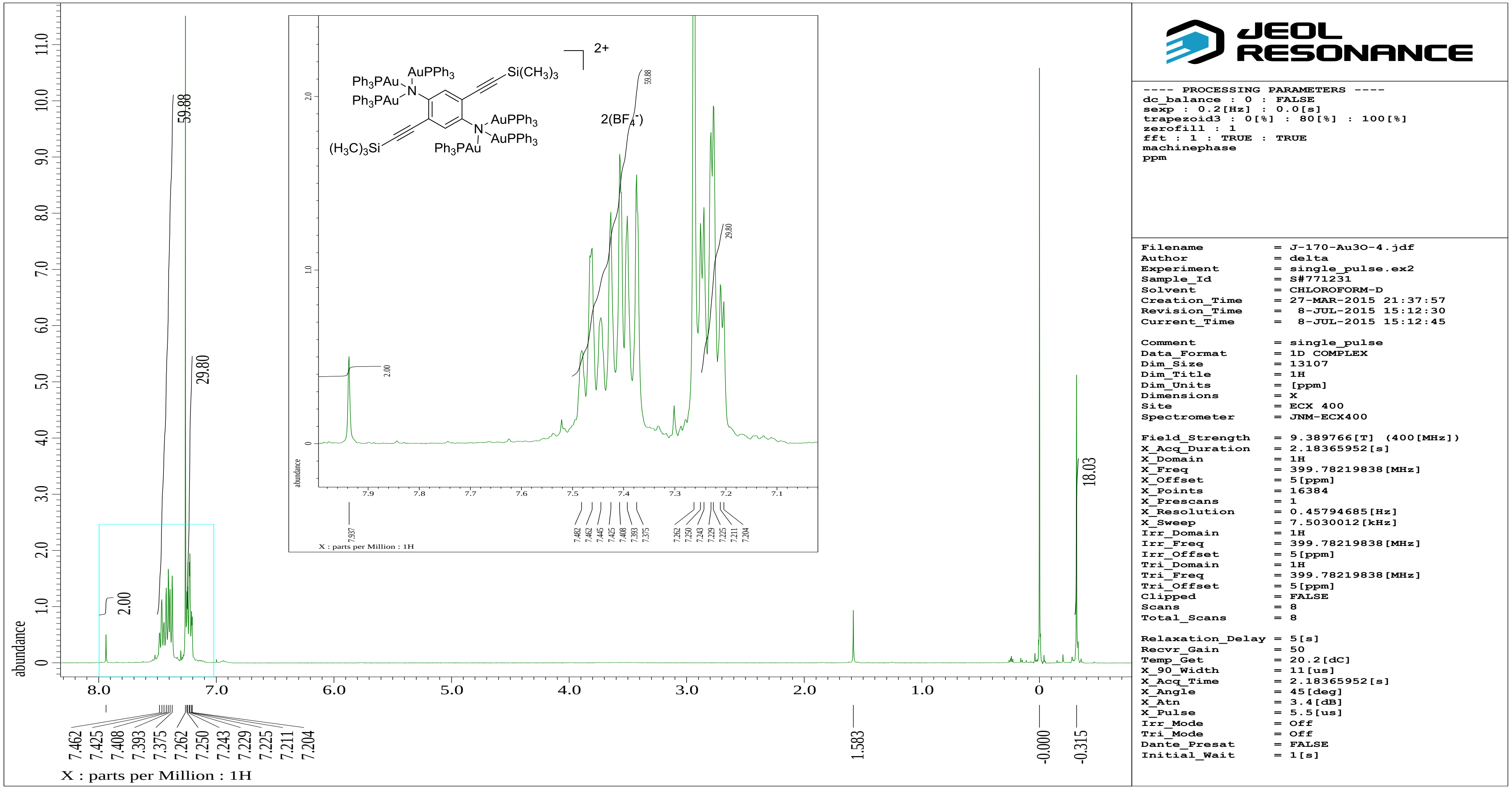


**Supplementary Figure 27.** $^{1}$H-NMR spectra of **9** in $CDCl_3$ at 298K.

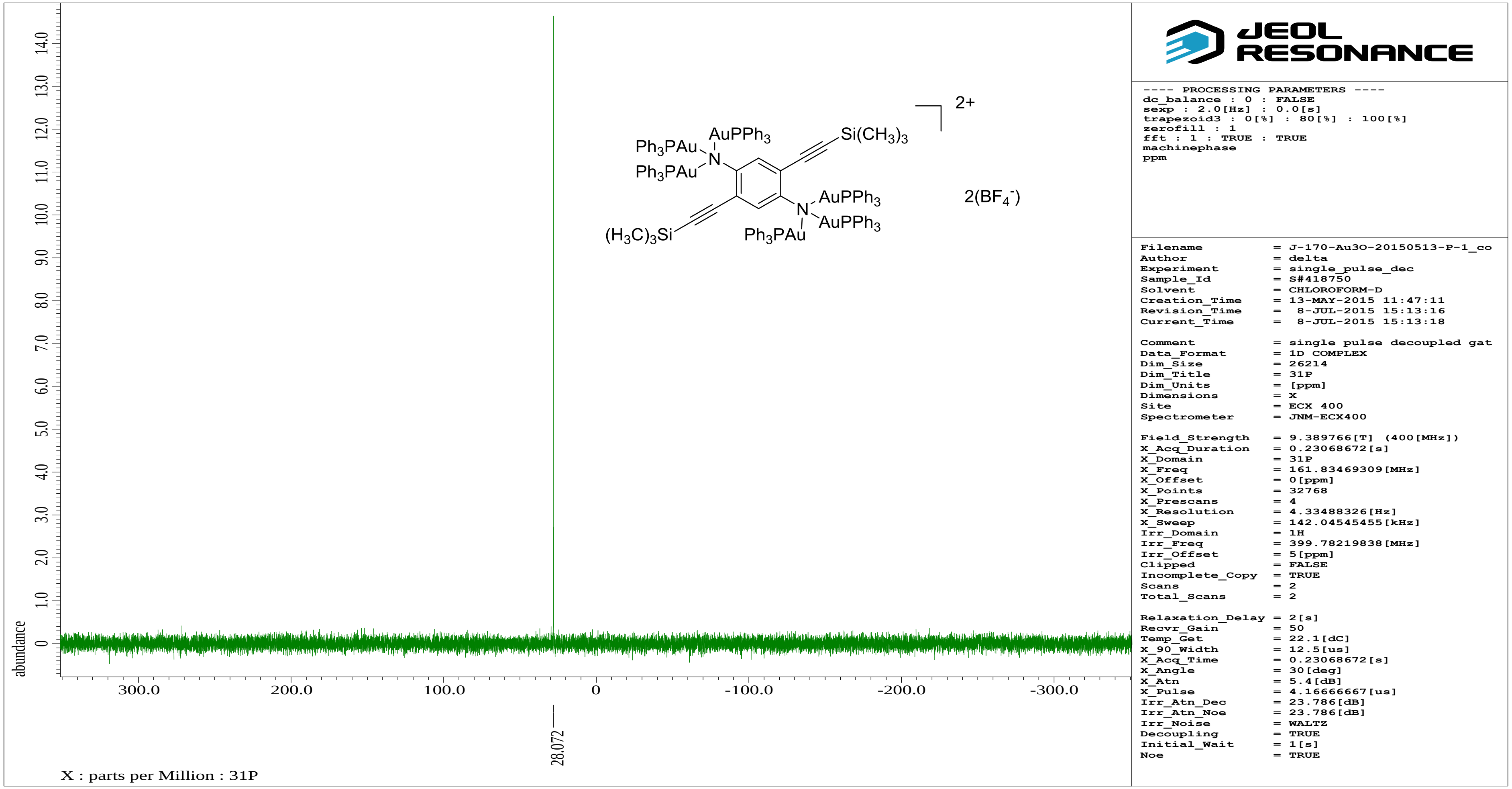


**Supplementary Figure 28.** $^{31}P$-NMR spectra of **9** in $CDCl_3$ at 298K.

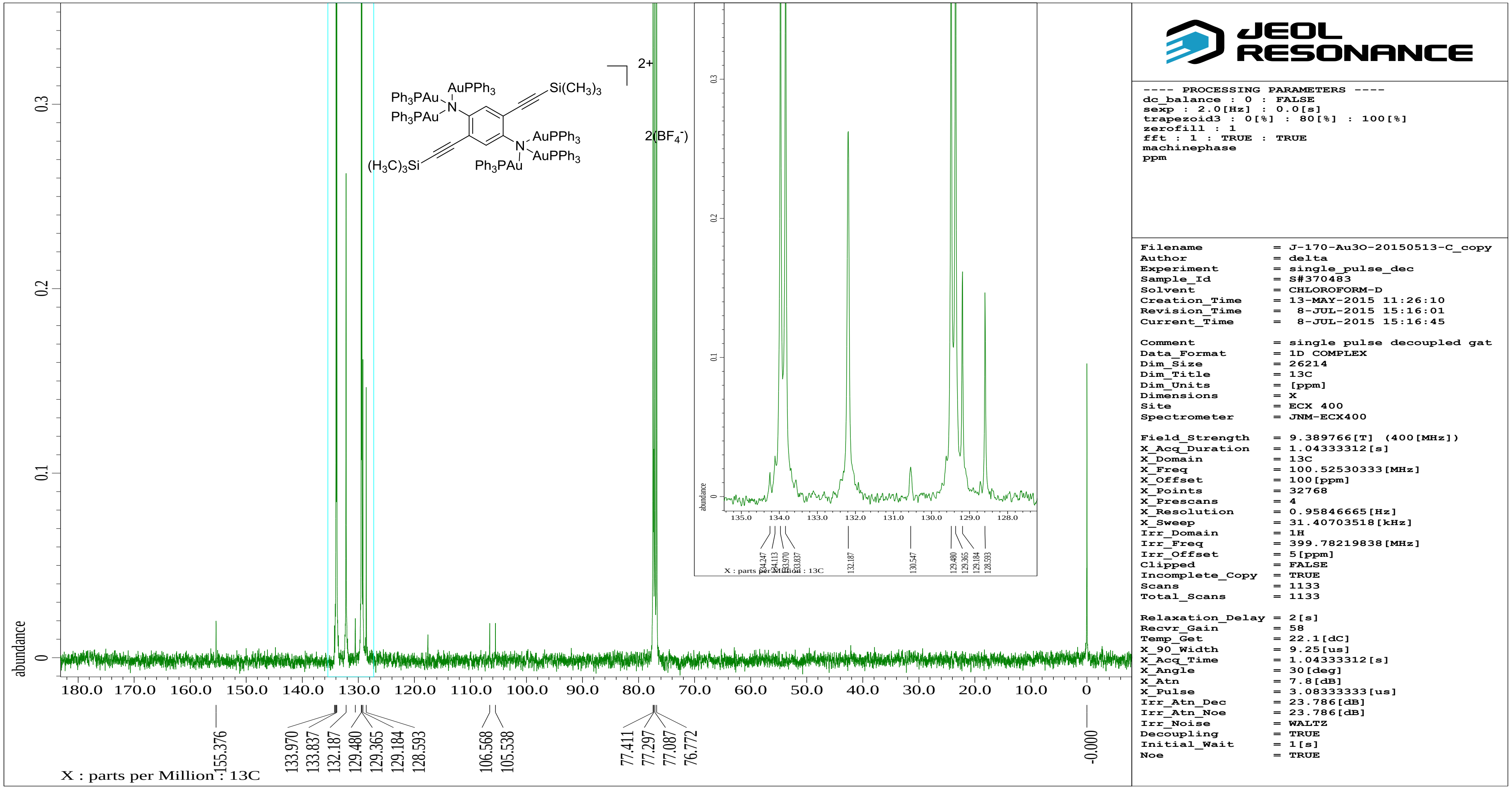


**Supplementary Figure 29.** $^{13}$C-NMR spectra of **9** in $CDCl_3$ at 298K.

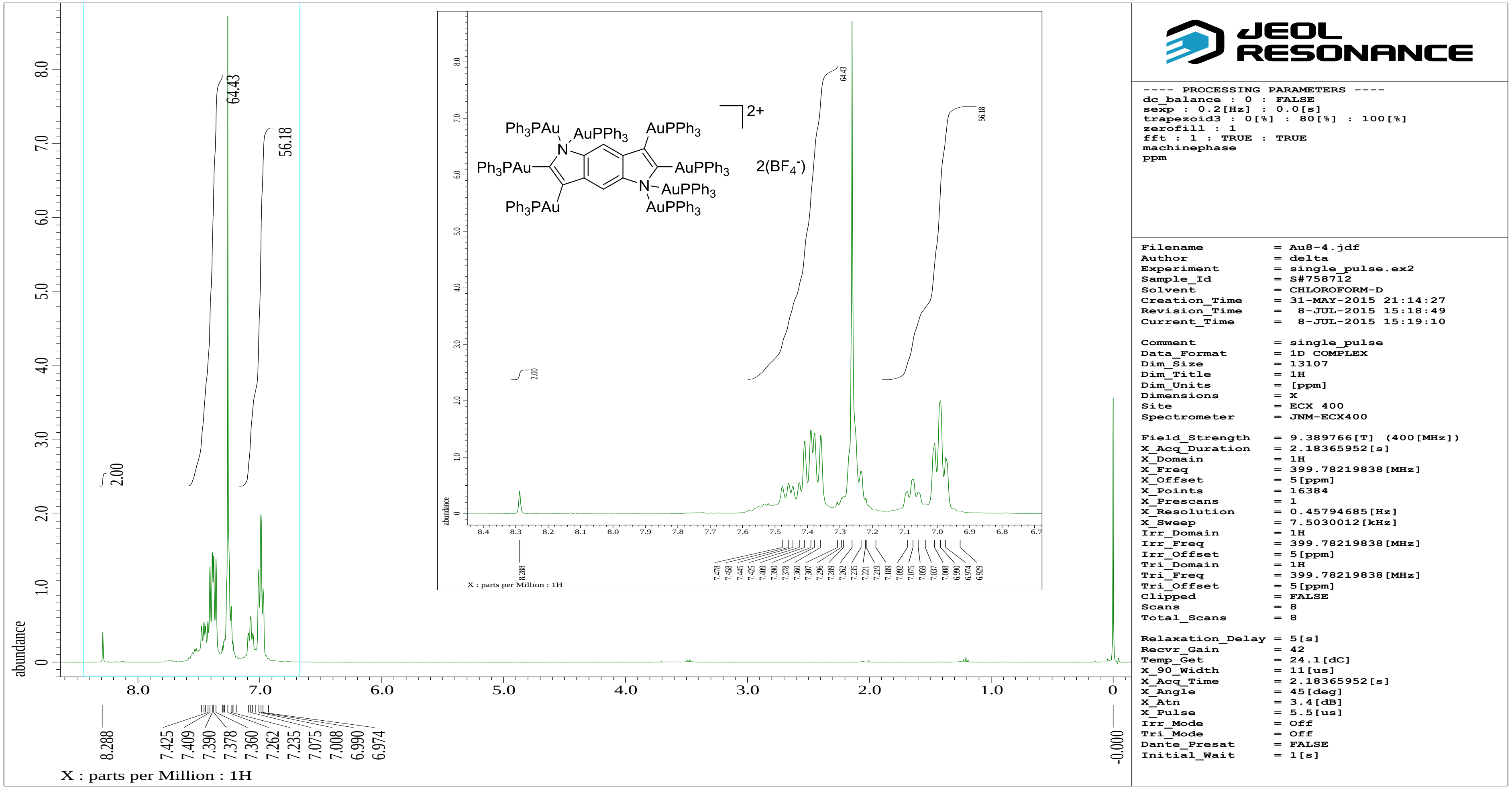


**Supplementary Figure 30.** $^{1}$H-NMR spectra of **10** in $CDCl_3$ at 298K.

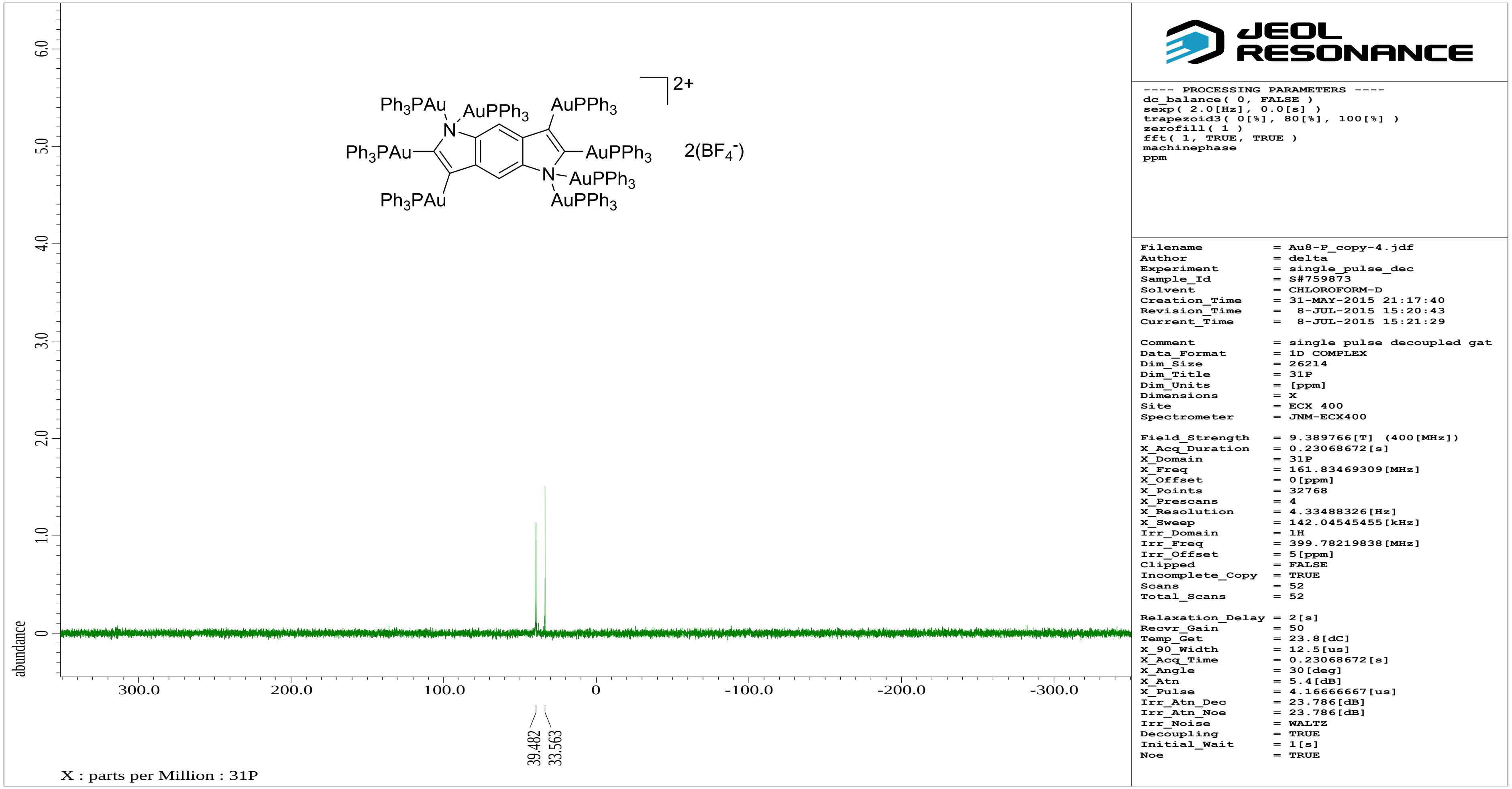


**Supplementary Figure 31.** $^{31}$P-NMR spectra of **10** in $CDCl_3$ at 298K.

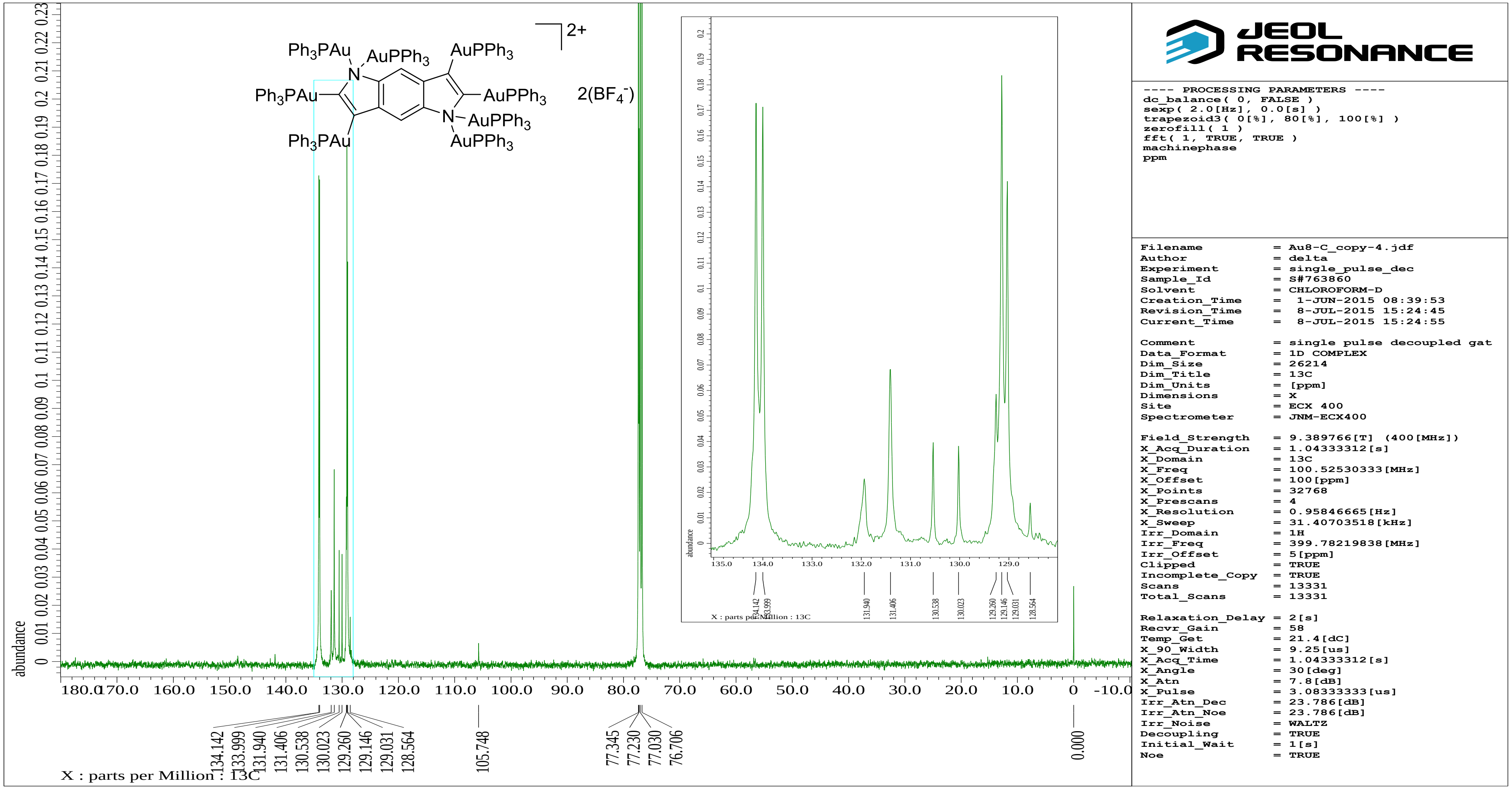


**Supplementary Figure 32.** $^{13}$C-NMR spectra of **10** in $CDCl_3$ at 298K.

## Supplementary Methods

All commercially available chemicals were used without further purification. 2-Ethynylaniline[1], 2-(2-phenylethynyl)benzenamine[1], oxotris((triphenylphosphine) gold) tetrafluoroborate[2] and 2,5-bis((trimethylsilyl)ethynyl)benzene-1,4-diamine[3,4] were synthesized according to the literature. Solvents used in this study were processed by standard procedures.

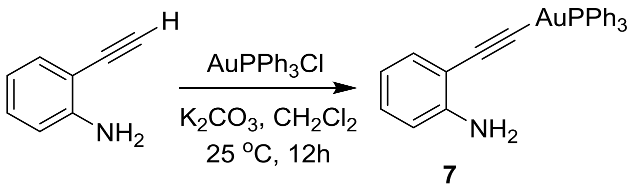


**7**

**Synthesis of 7.** 2-Ethynylaniline (22.4 mg, 0.19 mmol) and $AuPPh_3Cl$ (64.4 mg, 0.13 mmol) were dissolved in dry $CH_2Cl_2$ (2 ml). $K_2CO_3$ (500 mg, 3.62 mmol) was then added to the solution under stirring at room temperature for 12 hours. After being filtered through celite, the filtrate was concentrated under reduced pressure to yield product as a white solid. Yield: 88% (66 mg, 0.12 mmol). Single crystals of complex **7** were obtained by vapor diffusion of diethyl ether into a $CHCl_3$ solution of **7** after 2 days. $^1$H-NMR (400 MHz, $CDCl_3$): δ 7.58–7.44 (m, 15H), 7.35 (d, $J$ = 7.5 Hz, 1H), 7.35 (d, $J$ = 7.5 Hz, 1H), 7.05–6.99 (m, 1H), 6.70–6.60 (m, 2H), 6.70–6.59 (m, 2H), 4.38 (s, 2H). $^{13}$C-NMR (100 MHz, $CDCl_3$): δ 148.4, 134.4, 134.3, 132.4, 131.6, 130.0, 129.5, 129.2, 129.1, 128.0, 117.4, 113.7, 110.2. $^{31}$P-NMR (162 MHz, $CDCl_3$): δ 42.88. IR (KBr, $cm^{-1}$): 3457, 3353, 3069, 3052, 2103, 1968, 1896, 1812, 1608, 748, 710, 693. HR-MS (ESI): calcd. for $[M+H]^+$ ($C_{26}H_{22}AuNP$) 576.1150, found 576.11481. Elemental Analysis: Calcd. for $C_{26}H_{21}AuNP$: C, 54.27; H, 3.68; N, 2.43. Found: C, 54.92; H, 3.84; N, 2.57.

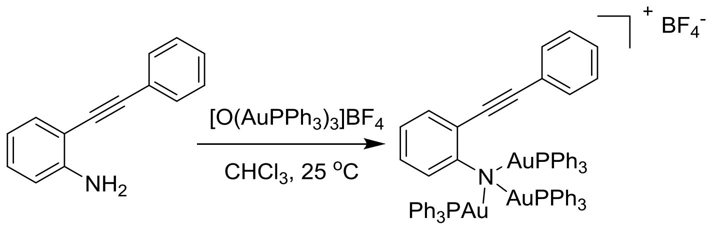


**5** **6**

**Synthesis of 6.** 2-(2-Phenylethynyl)benzenamine (**5**, 5.8 mg, 0.030 mmol) and oxotris((triphenylphosphine)gold)tetrafluoroborate (44.5 mg, 0.030 mmol) were dissolved in $CHCl_3$ (2 ml) with stirring at room temperature over 1.5 hours. The addition of hexane into the reaction mixture afforded a white solid of **6**. Yield: 92%

(46 mg, 0.028 mmol). Single crystals of **6** were obtained by vapor diffusion of diethyl ether into a $CHCl_3$ solution of **6**. $^1$H-NMR (400 MHz, $CDCl_3$): δ 7.85 (d, *J* = 8.0 Hz, 1H), 7.57 (d, *J* = 7.6 Hz, 1H), 7.43 (t, *J* = 7.3 Hz, 10H), 7.35 (dd, *J* = 13.0, 7.4 Hz, 20H), 7.23 (d, *J* = 7.4 Hz, 1H), 7.19–7.14 (m, 15H), 7.08 (d, *J* = 7.3 Hz, 2H), 7.01 (t, *J* = 7.5 Hz, 1H), 6.88 (t, *J* = 7.4 Hz, 1H), 6.72 (t, *J* = 7.7 Hz, 2H). $^{13}$C-NMR (100 MHz, $CDCl_3$): δ 162.3, 134.0, 133.8, 133.5, 132.2, 132.1, 131.3, 129.5, 129.3, 129.3, 129.2, 128.6, 128.1, 126.1, 123.0, 121.3, 117.3, 117.2, 99.9, 90.6. $^{31}$P-NMR (162 MHz, $CDCl_3$): δ 28.10. IR (KBr, $cm^{-1}$): 3055, 2200, 1968, 1895, 1813, 1609, 748, 711, 692; HR-MS (ESI): calcd. for $[M–BF_4]^+$ ($C_{68}H_{54}Au_3NP_3$) 1568.2460, found 1568.2458. Elemental analysis: Calcd. for ($C_{68}H_{54}Au_3BF_4NP_3$ + 1/2$CHCl_3$): C, 48.81; H, 3.58; N, 0.80. Found: C, 48.97; H, 3.22; N, 0.93.

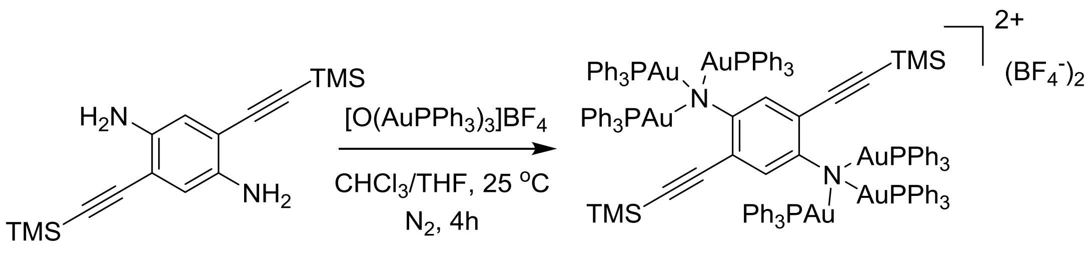


**8** **9**

**Synthesis of 9.** 2,5-bis((trimethylsilyl)ethynyl)benzene-1,4-diamine (**8**, 9 mg, 0.030 mmol) was dissolved in a mixed solution of dry $CHCl_3$ (2 ml) and dry THF (4 ml). Oxotris((triphenylphosphine)gold)tetrafluoroborate (90 mg, 0.060 mmol) was added to the solution under stirring at room temperature for 4 hours. Product **9** as a yellow solid precipitated from the solvent. Yield: 95% (91mg, 0.028 mmol). Single crystals of **9** were obtained by vapor diffusion of diethyl ether into a $CHCl_3$ solution of **9** after 1 day. $^1$H-NMR (400 MHz, $CDCl_3$): δ 7.94 (s, 2H), 7.48–7.37 (m, 60H), 7.25–7.21 (m, 30H), 0.32 (s, 18H). $^{13}$C-NMR (100MHz, $CDCl_3$): δ 155.6, 134.2, 134.0, 132.4, 132.4, 130.8, 129.7, 129.6, 129.4, 128.8, 117.8, 106.8, 105.8. $^{31}$P-NMR (162 MHz, $CDCl_3$): δ 28.07. IR (KBr, $cm^{-1}$): 3073, 3054, 2198, 2135, 1969, 1895, 1814, 1615, 1586, 749, 711, 693; HR-MS (ESI): calcd. for $[M–2BF_4]^{2+}$ ($C_{124}H_{110}Au_6N_2P_6Si_2$) 1525.7323, found 1525.7316. Elemental analysis: Calcd. for ($C_{124}H_{110}Au_6B_2F_8N_2P_6Si_2$ + 1/2$CHCl_3$): C, 45.98; H, 3.62; N, 0.84. Found: C, 45.75; H, 3.42; N, 0.86.

**X-ray crystallographic analysis**

Data for complexes **2**, **6**, **9** and **10** were collected at 173K with Mo-Kα radiation (λ = 0.71073 Å) on a Rigaku Saturn 724+ CCD diffractometer with frames of oscillation range 0.5°. All structures were solved by direct methods, and non-hydrogen atoms

were located from difference Fourier maps. All non-hydrogen atoms were subjected to anisotropic refinement by full-matrix least-squares on $F^2$ by using the SHELXTL program unless otherwise noticed[5]. All figures were drawn by using X-seed program[6].

Crystal data for **$[C_8H_4N(AuPPh_3)_4]BF_4{\cdot}CHCl_3$ (2)** (CCDC-1435042): $C_{81}H_{65}Au_4BCl_3F_4NP_4$, $M$ = 2157.25, monoclinic, space group $P2(1)/n$ (No. 14), $a$ = 17.579(4) Å, $b$ = 17.158(3) Å, $c$ = 25.460(5) Å, β = 101.80(3) °, $V$ = 7517(3) Å$^3$, $Z$ = 4, $T$ = 173(2) K, $D_c$ = 1.906 g/cm$^{-3}$. The structure, refined on $F^2$, converged for 13083 unique reflections ($R_{\rm int}$ = 0.0882) and 10544 observed reflections with I > 2σ(I) to give $R_1$ = 0.1162 and $wR_2$ = 0.3049 and a goodness-of-fit = 1.205.

Crystal data for **$[(C_6H_5C{\equiv}C)C_6H_4N(AuPPh_3)_3]BF_4{\cdot}CHCl_3$ (6)** (CCDC-1435041): $C_{138}H_{110}Au_6B_2Cl_6F_8N_2P_6$, $M$ = 3550.22, triclinic, space group $P$-1 (No. 2), $a$ = 17.519(4) Å, $b$ = 19.670(4) Å, $c$ = 20.326(4) Å, $\alpha$ = 76.07(3) °, $\beta$ = 81.79(3) °, $\gamma$ = 72.44(3) °, $V$ = 6463(2) Å$^3$, $Z$ = 2, $T$ = 173(2) K, $D_c$ = 1.824 g/cm$^{-3}$. The structure, refined on $F^2$, converged for 29563 unique reflections ($R_{\rm int}$ = 0.0726) and 23826 observed reflections with I > 2σ(I) to give $R_1$ = 0.0755 and $wR_2$ = 0.2037 and a goodness-of-fit = 1.158.

Crystal data for **$[C_6H_2(C{\equiv}CSiMe_3)_2N_2(AuPPh_3)_6](BF_4)_2{\cdot}2CHCl_3$ (9)** (CCDC-1435040): $C_{63}H_{56}Au_3BCl_3F_4NP_3Si$, $M$ = 1732.15, triclinic, space group $P$-1 (No. 2), $a$ = 10.506(2) Å, $b$ = 16.748(3) Å, $c$ = 19.375(4) Å, $\alpha$ = 67.55(3) °, $\beta$ = 89.98(3) °, $\gamma$ = 89.81(3) °, $V$ = 3150.7(11) Å$^3$, $Z$ = 2, $T$ = 173(2) K, $D_c$ = 1.826 g/cm$^{-3}$. The structure, refined on $F^2$, converged for 11087 unique reflections ($R_{\rm int}$ = 0.1111) and 10099 observed reflections with I > 2σ(I) to give $R_1$ = 0.0579 and $wR_2$ = 0.1490 and a goodness-of-fit = 1.092.

Crystal data for **$[C_{10}H_2N_2(AuPPh_3)_8](BF_4)_2{\cdot}2CHCl_3$ (10)** (CCDC-1435045): $C_{156}H_{124}Au_8B_2Cl_6F_8N_2P_8$, M = 4236.38, monoclinic, space group $P2(1)/n$ (No. 14), $a$ = 13.435(3) Å, $b$ = 15.565(3) Å, $c$ = 35.747(7) Å, $\beta$ = 97.59(3) °, $V$ = 7410(3) Å$^3$, $Z$ = 2, $T$ = 173(2) K, $D_c$ = 1.899 g/cm$^{-3}$. The structure, refined on $F^2$, converged for 16984 unique reflections ($R_{\rm int}$ = 0.1046) and 14871 observed reflections with I > 2σ(I) to give $R_1$ = 0.0755 and $wR_2$ = 0.2067 and a goodness-of-fit = 1.161. The fluorine atoms of the $BF_4$ moiety are disordered at two positions (F1, F2, F3 and F4) and (F1', F2', F3' and F4') with a refined site-occupancy ratio of 0.69:0.31.

**Computational details.** All structures were optimized at the TPSS[7] level of density functional theory with "EmpiricalDispersion=GD3"[8] to describe the dispersion corrections. In addition, the frequency calculations were performed to confirm the

characteristics of the calculated structures as minima. In the calculations, the fully relativistic effective core potentials (ECPs) with ECP60MDF[9] and ECP46MDF[10] basis set were used to describe the heavy atoms, Au and Sn, respectively, whereas the 6-31G(d) basis set for the C, N, P, and H atoms[11]. All the optimizations were performed with the Gaussian 09 software package[12]. The calculated Au···Au distance (2.889 Å) and Au-N bond lengths (2.132 and 2.120 Å) in the model complex **2A** are close to the experimental values (2.862, 2,166 and 2.082 Å, respectively), indicating the reliability of our calculations. TD-DFT calculations were performed on model complexes **2'** and **10'** using the PBE1PBE functional[16], the PCM model with dichloromethane as the solvent and NStates = 20. The NICS values were calculated at the TPSS-GIAO level.

**Cartesian coordinates together with the symmetry and electronic energies for all the complexes calculated in this study.**

**2'** ($S_0$)

E = -2277.539582 a.u.

| | | | |
|---|---|---|---|
| Au | 1.97755000 | 0.25231000 | -1.62775800 |
| Au | 1.97630800 | 0.25190600 | 1.62836800 |
| Au | -0.45362600 | -1.84089400 | -0.00049700 |
| Au | -3.42450200 | 0.50586000 | -0.00078600 |
| N | 0.85814200 | 0.92127500 | -0.00006300 |
| C | -0.42531300 | 0.17733100 | -0.00048800 |
| C | -1.47909800 | 1.07307400 | -0.00041800 |
| C | -0.91072900 | 2.41588800 | 0.00000500 |
| C | -1.49444900 | 3.69935500 | 0.00016400 |
| H | -2.57890100 | 3.80262500 | 0.00000100 |
| C | -0.66365300 | 4.82186300 | 0.00050500 |
| H | -1.10341200 | 5.81796300 | 0.00062700 |
| C | 0.74413300 | 4.69169100 | 0.00066400 |
| H | 1.36705900 | 5.58444900 | 0.00089800 |
| C | 1.34796500 | 3.42923200 | 0.00050000 |
| H | 2.43214900 | 3.31398200 | 0.00060100 |
| C | 0.50155700 | 2.31498300 | 0.00018700 |
| P | 3.16671700 | -0.41170100 | -3.41127800 |
| P | 3.16414700 | -0.41231400 | 3.41268700 |

| | | | |
|---|---|---|---|
| P | -0.56605400 | -4.14286400 | 0.00006000 |
| P | -5.64391500 | -0.11269200 | 0.00059400 |
| H | -1.20640400 | -4.79112100 | 1.07695900 |
| H | 0.64025600 | -4.87274200 | -0.00991400 |
| H | -1.22404200 | -4.79081600 | -1.06634700 |
| H | -6.10687100 | -1.02003000 | 0.97692900 |
| H | -6.18818100 | -0.73130400 | -1.14466300 |
| H | -6.60309000 | 0.90554000 | 0.17588400 |
| H | 3.59527800 | 0.59468600 | 4.29544900 |
| H | 2.54558800 | -1.29614500 | 4.31688300 |
| H | 4.37768600 | -1.08941700 | 3.18660300 |
| H | 4.37993200 | -1.08911300 | -3.18438300 |
| H | 3.59876300 | 0.59541100 | -4.29346700 |
| H | 2.54868500 | -1.29519700 | -4.31616100 |

**2'** ($T_1$)

E = -2277.492980 a.u.

| | | | |
|---|---|---|---|
| Au | 2.15827100 | -1.09134800 | -0.79580700 |
| Au | 2.11630500 | 1.43573200 | 0.61925200 |
| Au | -0.18290900 | -1.25121800 | 0.69140500 |
| Au | -3.69111600 | -0.12003400 | -0.16095700 |
| N | 0.12967900 | 1.67624400 | 0.14807500 |
| C | -0.74246100 | 0.63816500 | 0.19403200 |
| C | -2.08649200 | 1.09786700 | -0.15190900 |
| C | -1.95138500 | 2.50496200 | -0.41339900 |
| C | -2.86956500 | 3.50775000 | -0.78444900 |
| H | -3.91955700 | 3.25948700 | -0.93365800 |
| C | -2.40463000 | 4.82250200 | -0.95416800 |
| H | -3.09831200 | 5.60994400 | -1.24133100 |
| C | -1.04875400 | 5.13319400 | -0.75620000 |
| H | -0.70813200 | 6.15803900 | -0.89229400 |
| C | -0.10956100 | 4.14044300 | -0.38033100 |
| H | 0.94019700 | 4.38615300 | -0.22390100 |
| C | -0.58110500 | 2.84754300 | -0.21727800 |
| P | 3.39250900 | -1.64933600 | -2.70451100 |

| | | | |
|---|---|---|---|
| P | 4.28212200 | 1.34365100 | 1.23607200 |
| P | 0.26045300 | -3.42872900 | 1.37277200 |
| P | -5.54097300 | -1.51226200 | -0.14712200 |
| H | -0.70330700 | -4.22322500 | 2.03634600 |
| H | 1.33561500 | -3.59463300 | 2.26674500 |
| H | 0.64570600 | -4.34262900 | 0.37241600 |
| H | -5.81250900 | -2.25300600 | 1.02072100 |
| H | -5.61562400 | -2.54880900 | -1.09929400 |
| H | -6.80515500 | -0.92367500 | -0.34681300 |
| H | 4.91537700 | 2.45763000 | 1.82811600 |
| H | 4.61370900 | 0.34761600 | 2.17287000 |
| H | 5.21208600 | 1.04988100 | 0.22114400 |
| H | 4.41923600 | -2.60682300 | -2.55931700 |
| H | 4.13515000 | -0.64122400 | -3.35804600 |
| H | 2.74636100 | -2.20031800 | -3.83372500 |

**10'** ($S_0$)

E = -4322.746590 a.u.

| | | | |
|---|---|---|---|
| Au | 3.44410900 | 1.96011500 | 1.57292400 |
| Au | 3.44399300 | 1.96058500 | -1.57260700 |
| Au | 5.37429600 | -0.58928700 | -0.00015100 |
| Au | 2.96239800 | -3.45715700 | 0.00035300 |
| N | 2.66474000 | 0.82616600 | 0.00000800 |
| C | 3.35581800 | -0.48332700 | 0.00002200 |
| C | 2.42079800 | -1.50272600 | -0.00001700 |
| C | 1.10146600 | -0.88586300 | -0.00006000 |
| C | 1.25652100 | 0.53456700 | -0.00000300 |
| C | -0.19390500 | -1.43673900 | -0.00004900 |
| H | -0.34887600 | -2.51571800 | -0.00006700 |
| P | 4.27080100 | 3.16180800 | 3.28217600 |
| P | 4.27047200 | 3.16285100 | -3.28156500 |
| P | 7.67443800 | -0.78610700 | -0.00077900 |
| P | 3.62490800 | -5.66563300 | -0.00060200 |
| N | -2.66470700 | -0.82618700 | 0.00000300 |
| C | -3.35578400 | 0.48330700 | 0.00001700 |

| | | | |
|---|---|---|---|
| C | -2.42076400 | 1.50270600 | 0.00005500 |
| C | -1.10143400 | 0.88584000 | 0.00007600 |
| C | -1.25649000 | -0.53459000 | 0.00001500 |
| C | 0.19393600 | 1.43671700 | 0.00006400 |
| H | 0.34890700 | 2.51569600 | 0.00008300 |
| Au | -3.44411800 | -1.95998600 | -1.57300900 |
| Au | -3.44402400 | -1.96071300 | 1.57250300 |
| Au | -5.37426000 | 0.58929200 | 0.00025100 |
| Au | -2.96236500 | 3.45713600 | -0.00025800 |
| P | -4.27087800 | -3.16150200 | -3.28235200 |
| P | -4.27060400 | -3.16309400 | 3.28133100 |
| P | -7.67439600 | 0.78618800 | 0.00095800 |
| P | -3.62492200 | 5.66559800 | 0.00076100 |
| H | 4.95578200 | 4.35384300 | 2.98031000 |
| H | 5.20901300 | 2.53072900 | 4.11966000 |
| H | 3.34799700 | 3.62941100 | 4.23544000 |
| H | 4.95454000 | 4.35535300 | -2.97947800 |
| H | 3.34767200 | 3.62982100 | -4.23513900 |
| H | 5.20939900 | 2.53241100 | -4.11873100 |
| H | 8.44417100 | 0.39455900 | 0.00492300 |
| H | 8.29303000 | -1.46397800 | 1.06953600 |
| H | 8.29322900 | -1.45366200 | -1.07745300 |
| H | 4.25664000 | -6.18869300 | 1.14677600 |
| H | 4.54788300 | -6.09683100 | -0.97591100 |
| H | 2.63774900 | -6.65654300 | -0.17895400 |
| H | -3.34793200 | -3.62970800 | 4.23520300 |
| H | -5.20995400 | -2.53286800 | 4.11818400 |
| H | -4.95423000 | -4.35582400 | 2.97915300 |
| H | -4.95498200 | -4.35407800 | -2.98064600 |
| H | -3.34826300 | -3.62814400 | -4.23626500 |
| H | -5.20989000 | -2.53065100 | -4.11911100 |
| H | -8.44418100 | -0.39444200 | -0.00518300 |
| H | -8.29297000 | 1.46449200 | -1.06909300 |
| H | -8.29314900 | 1.45335700 | 1.07789200 |
| H | -4.25675800 | 6.18864900 | -1.14656300 |

| | | | |
|---|---|---|---|
| H | -4.54782400 | 6.09677300 | 0.97614900 |
| H | -2.63776900 | 6.65652800 | 0.17903800 |

**2A** ($S_0$)

E= -2707.628613 a.u.

| | | | |
|---|---|---|---|
| Au | 1.49709400 | 1.03341000 | -0.49319400 |
| Au | -1.39075800 | 0.98351400 | -0.42125300 |
| N | 0.02491500 | 2.50874600 | -0.88352600 |
| C | -0.03540200 | 3.04828400 | -2.20836700 |
| C | -0.08640000 | 4.41289000 | -2.17896900 |
| C | -0.05936400 | 4.82452900 | -0.79258000 |
| C | -0.08704500 | 6.07392000 | -0.14040000 |
| H | -0.14016600 | 6.99696900 | -0.71528200 |
| C | -0.04421900 | 6.10370400 | 1.25528700 |
| H | -0.06409000 | 7.06140700 | 1.77243400 |
| C | 0.02531900 | 4.91282400 | 2.01424800 |
| H | 0.05971700 | 4.97032900 | 3.10074200 |
| C | 0.05311700 | 3.66249000 | 1.38850700 |
| H | 0.10925900 | 2.73531300 | 1.96043600 |
| C | 0.01034300 | 3.64272800 | -0.01114800 |
| P | 2.89625300 | -0.66357800 | 0.03615700 |
| C | 4.47245000 | -0.15679900 | 0.79946700 |
| C | 5.04617800 | 1.07153600 | 0.41413000 |
| H | 4.52681400 | 1.71443500 | -0.29665000 |
| C | 6.27715300 | 1.46486900 | 0.95156400 |
| H | 6.71750900 | 2.41503200 | 0.65500500 |
| C | 6.93412300 | 0.64003700 | 1.87635800 |
| H | 7.88845900 | 0.95040500 | 2.29815800 |
| C | 6.36135700 | -0.57935600 | 2.26711100 |
| H | 6.86881000 | -1.21478400 | 2.99077300 |
| C | 5.13116100 | -0.98209100 | 1.73285900 |
| H | 4.68124800 | -1.92356400 | 2.04504600 |
| C | 3.29281800 | -1.72523200 | -1.39915100 |
| C | 2.24375500 | -2.05539100 | -2.28227500 |
| H | 1.24727500 | -1.64369300 | -2.12196400 |

| | | | |
|---|---|---|---|
| C | 2.48190500 | -2.91958800 | -3.35582800 |
| H | 1.66785900 | -3.17681800 | -4.03149000 |
| C | 3.76710400 | -3.44508700 | -3.56162700 |
| H | 3.95349900 | -4.11099500 | -4.40242300 |
| C | 4.81329000 | -3.10851000 | -2.69140900 |
| H | 5.81130000 | -3.51131000 | -2.85480900 |
| C | 4.58111400 | -2.25123400 | -1.60654100 |
| H | 5.39445500 | -1.99133900 | -0.93095500 |
| C | 2.04359600 | -1.77393600 | 1.22223200 |
| C | 2.21102800 | -3.17163300 | 1.16773000 |
| H | 2.88089200 | -3.61247800 | 0.43065900 |
| C | 1.49653200 | -3.99493000 | 2.04925200 |
| H | 1.62390200 | -5.07504600 | 1.99980400 |
| C | 0.61420000 | -3.43131100 | 2.98150600 |
| H | 0.04700600 | -4.07379900 | 3.65261300 |
| C | 0.45208300 | -2.03943400 | 3.04615000 |
| H | -0.24365600 | -1.60114600 | 3.75825900 |
| C | 1.16283800 | -1.21182300 | 2.17053900 |
| H | 1.01861200 | -0.13197400 | 2.20593700 |
| P | -2.84104700 | -0.68275600 | 0.06711500 |
| C | -4.56492700 | -0.33424200 | -0.42540300 |
| C | -4.80445700 | 0.54748400 | -1.49831900 |
| H | -3.96795400 | 1.04531400 | -1.98907300 |
| C | -6.11575300 | 0.78774000 | -1.92542100 |
| H | -6.29808900 | 1.47192600 | -2.75211400 |
| C | -7.18964300 | 0.15645200 | -1.28156800 |
| H | -8.20924700 | 0.35020300 | -1.60997600 |
| C | -6.95460800 | -0.71742100 | -0.20956800 |
| H | -7.78915700 | -1.20187000 | 0.29417900 |
| C | -5.64606200 | -0.96627900 | 0.22112300 |
| H | -5.46653600 | -1.63898200 | 1.05827400 |
| C | -2.35582900 | -2.23798700 | -0.77000000 |
| C | -3.23782900 | -2.94172400 | -1.61005200 |
| H | -4.24905100 | -2.57404300 | -1.77457100 |
| C | -2.80688800 | -4.11921600 | -2.23820400 |

| | | | |
|---|---|---|---|
| H | -3.49013200 | -4.66135500 | -2.88965100 |
| C | -1.50612600 | -4.59683500 | -2.02817700 |
| H | -1.17780600 | -5.51227600 | -2.51776200 |
| C | -0.62735400 | -3.89704000 | -1.18650000 |
| H | 0.38486900 | -4.25976300 | -1.01930700 |
| C | -1.04549000 | -2.71748600 | -0.56243600 |
| H | -0.36272100 | -2.17496800 | 0.08998300 |
| C | -2.87265600 | -1.07533300 | 1.85555000 |
| C | -2.75920500 | -0.02354600 | 2.78696300 |
| H | -2.61994800 | 0.99984100 | 2.43738300 |
| C | -2.82734600 | -0.29441000 | 4.15905300 |
| H | -2.74191300 | 0.52037300 | 4.87594200 |
| C | -3.00547300 | -1.61302600 | 4.60727000 |
| H | -3.05925700 | -1.82128100 | 5.67447300 |
| C | -3.11077200 | -2.66196000 | 3.68209100 |
| H | -3.24618700 | -3.68523200 | 4.02852100 |
| C | -3.04230500 | -2.39828000 | 2.30749700 |
| H | -3.10992300 | -3.21516100 | 1.59077200 |
| H | -0.03092400 | 2.36219600 | -3.04736800 |
| H | -0.13563300 | 5.06489100 | -3.04434700 |

**2B** ($S_0$)

E = -2707.570887 a.u.

| | | | |
|---|---|---|---|
| N | 1.21447500 | -0.34168600 | 0.00000000 |
| C | -0.06873300 | -1.25889400 | 0.00118400 |
| C | -1.14616300 | -0.42632000 | -0.00006900 |
| C | -0.70113800 | 0.97870400 | -0.00044300 |
| C | -1.41988900 | 2.18390700 | 0.00109800 |
| H | -2.50831800 | 2.16677700 | 0.00359400 |
| C | -0.71118000 | 3.39455100 | 0.00126600 |
| H | -1.25837600 | 4.33565000 | 0.00309800 |
| C | 0.69463400 | 3.42168300 | -0.00015500 |
| H | 1.21955300 | 4.37446000 | 0.00029100 |
| C | 1.43601500 | 2.22545000 | -0.00109400 |
| H | 2.52479500 | 2.23702600 | -0.00145600 |

| | | | |
|---|---|---|---|
| C | 0.70155900 | 1.04819600 | -0.00067200 |
| P | -0.32920600 | -5.57123500 | 0.15564000 |
| C | -0.48023800 | -6.36040700 | -1.49268400 |
| C | -1.13410100 | -5.64412900 | -2.51597100 |
| H | -1.49247800 | -4.63182000 | -2.32785400 |
| C | -1.33076400 | -6.23627900 | -3.76905800 |
| H | -1.84218500 | -5.68089400 | -4.55347500 |
| C | -0.86652900 | -7.53773600 | -4.01107100 |
| H | -1.01373000 | -7.99468000 | -4.98830600 |
| C | -0.20545900 | -8.24861900 | -2.99878800 |
| H | 0.16065700 | -9.25631400 | -3.18833900 |
| C | -0.01113000 | -7.66519700 | -1.73961400 |
| H | 0.50581700 | -8.21623200 | -0.95500400 |
| C | 0.99752500 | -6.49108900 | 1.01874500 |
| C | 2.33395900 | -6.19560400 | 0.67912300 |
| H | 2.54626700 | -5.42538900 | -0.06280000 |
| C | 3.38175100 | -6.89435200 | 1.28808800 |
| H | 4.41222900 | -6.66675000 | 1.02018900 |
| C | 3.10398200 | -7.88202100 | 2.24570500 |
| H | 3.92068000 | -8.42108800 | 2.72279700 |
| C | 1.77717200 | -8.17232000 | 2.59086800 |
| H | 1.56043100 | -8.93644300 | 3.33549500 |
| C | 0.72190000 | -7.48185400 | 1.97913500 |
| H | -0.30756300 | -7.70932700 | 2.25047700 |
| C | -1.89969100 | -5.97924400 | 1.01267000 |
| C | -2.60945900 | -7.16027600 | 0.71626000 |
| H | -2.22029800 | -7.85905200 | -0.02317600 |
| C | -3.82717300 | -7.42462600 | 1.35622800 |
| H | -4.37539500 | -8.33426600 | 1.11661900 |
| C | -4.34259000 | -6.51540100 | 2.29048700 |
| H | -5.29691800 | -6.71225600 | 2.77513200 |
| C | -3.63900100 | -5.34123300 | 2.59371400 |
| H | -4.04915000 | -4.62796400 | 3.30538100 |
| C | -2.42184900 | -5.07058800 | 1.95748100 |
| H | -1.88897400 | -4.14475000 | 2.17394900 |

| | | | |
|---|---|---|---|
| P | -5.18944800 | -2.05616100 | -0.05568400 |
| C | -5.77557200 | -2.66774500 | 1.57231400 |
| C | -5.32155000 | -2.01966700 | 2.73856800 |
| H | -4.61364800 | -1.19469200 | 2.65692700 |
| C | -5.77213600 | -2.43943900 | 3.99655700 |
| H | -5.41798700 | -1.93558300 | 4.89442500 |
| C | -6.67369300 | -3.51044500 | 4.09793400 |
| H | -7.02186300 | -3.83775100 | 5.07625000 |
| C | -7.12459400 | -4.16023100 | 2.93941500 |
| H | -7.82454100 | -4.99102100 | 3.01628700 |
| C | -6.67647600 | -3.74452700 | 1.67833900 |
| H | -7.01715800 | -4.25934300 | 0.78147400 |
| C | -5.41008600 | -3.47569300 | -1.19732600 |
| C | -6.47932500 | -3.54724600 | -2.11004000 |
| H | -7.20161100 | -2.73498500 | -2.17014100 |
| C | -6.60923800 | -4.66572200 | -2.94529100 |
| H | -7.43557700 | -4.71555700 | -3.65263500 |
| C | -5.68252200 | -5.71455200 | -2.86952600 |
| H | -5.78810600 | -6.58165200 | -3.51984400 |
| C | -4.61794100 | -5.64653300 | -1.95817800 |
| H | -3.89281000 | -6.45504500 | -1.89640400 |
| C | -4.47550100 | -4.52894900 | -1.12915900 |
| H | -3.64814500 | -4.47796300 | -0.42284000 |
| C | -6.42294400 | -0.80637200 | -0.58317000 |
| C | -7.72882700 | -0.77558100 | -0.05730900 |
| H | -8.02550700 | -1.48656800 | 0.71190300 |
| C | -8.64617300 | 0.17704700 | -0.51953100 |
| H | -9.65337700 | 0.19950100 | -0.10664500 |
| C | -8.26968900 | 1.09757000 | -1.50771300 |
| H | -8.98523000 | 1.83722700 | -1.86303900 |
| C | -6.97008100 | 1.06996700 | -2.03490200 |
| H | -6.67430200 | 1.78607300 | -2.79994500 |
| C | -6.04636900 | 0.12537500 | -1.57258500 |
| H | -5.03330500 | 0.10718900 | -1.97539500 |
| H | 1.78869200 | -0.55617000 | -0.82832100 |

| | | | |
|---|---|---|---|
| H | 1.79037200 | -0.55549300 | 0.82735200 |
| Au | -0.04067600 | -3.27913900 | 0.05153400 |
| Au | -3.03932100 | -1.19906400 | -0.01344700 |

**2C** ($S_0$)

E= -364.225559 a.u.

| | | | |
|---|---|---|---|
| N | -1.61106500 | -1.09737700 | 0.00002500 |
| C | -2.41961500 | 0.17147200 | -0.00005500 |
| C | -1.57990400 | 1.22174800 | 0.00003000 |
| C | -0.18903700 | 0.76335300 | 0.00000500 |
| C | 1.03747700 | 1.44036500 | -0.00000500 |
| H | 1.07692200 | 2.52693700 | 0.00000200 |
| C | 2.21357100 | 0.67347700 | -0.00000600 |
| H | 3.17720000 | 1.17763800 | -0.00000500 |
| C | 2.17623000 | -0.73184300 | -0.00000800 |
| H | 3.10541200 | -1.29651200 | -0.00001000 |
| C | 0.95287700 | -1.43054100 | -0.00000200 |
| H | 0.92193100 | -2.51804900 | 0.00000000 |
| C | -0.18415400 | -0.64016100 | 0.00000800 |
| H | -1.89884400 | 2.25896200 | 0.00004100 |
| H | -3.49780700 | 0.07631500 | -0.00010800 |
| H | -1.82602300 | -1.67539500 | 0.83071500 |
| H | -1.82601200 | -1.67547600 | -0.83061000 |

**2D** ($S_0$)

E= -562.554431 a.u.

| | | | |
|---|---|---|---|
| N | 1.35591400 | -0.19355800 | -0.00003900 |
| C | 1.71104400 | 1.23745300 | 0.00003200 |
| C | 0.55690200 | 1.93293000 | -0.00018400 |
| C | -0.59618300 | 1.01264300 | 0.00000400 |
| C | -1.97531900 | 1.20119400 | 0.00008900 |
| H | -2.40980500 | 2.19797300 | 0.00005800 |
| C | -2.79904100 | 0.04932200 | 0.00010900 |
| H | -3.87919700 | 0.17579900 | 0.00013300 |
| C | -2.26298000 | -1.24572400 | 0.00010100 |

| | | | |
|---|---|---|---|
| H | -2.92684600 | -2.10650000 | 0.00013400 |
| C | -0.86457600 | -1.45696700 | 0.00005700 |
| H | -0.42483900 | -2.45058900 | 0.00006000 |
| C | -0.10699600 | -0.30283000 | 0.00000000 |
| H | 0.49489100 | 3.01681100 | -0.00025400 |
| H | 2.76673200 | 1.47143800 | 0.00012100 |
| F | 1.93946300 | -0.86208800 | -1.09824200 |
| F | 1.93948800 | -0.86215200 | 1.09810700 |

**2E** ($S_0$)

E= -373.439590 a.u.

| | | | |
|---|---|---|---|
| N | 0.12239100 | -0.00000800 | 0.41668500 |
| C | -0.04897200 | -0.00016300 | 1.87631000 |
| C | -1.36569400 | -0.00022000 | 2.21113000 |
| C | -2.15360100 | -0.00030900 | 0.99404600 |
| C | -3.53828200 | -0.00049600 | 0.74285000 |
| H | -4.25114800 | -0.00052900 | 1.56447200 |
| C | -3.97189700 | -0.00063500 | -0.58568700 |
| H | -5.03804100 | -0.00077800 | -0.80138200 |
| C | -3.05444200 | -0.00060100 | -1.65709900 |
| H | -3.42338100 | -0.00070300 | -2.68027600 |
| C | -1.67048300 | -0.00043200 | -1.43100800 |
| H | -0.96875400 | -0.00039100 | -2.26383800 |
| C | -1.25203200 | -0.00025700 | -0.09579100 |
| H | -1.75371100 | -0.00025800 | 3.22406100 |
| H | 0.83846500 | -0.00007700 | 2.50139700 |
| Sn | 1.08589100 | 1.88554000 | -0.15337600 |
| H | 2.54554000 | 1.78273100 | 0.68000900 |
| H | 1.21427500 | 1.72045700 | -1.82335900 |
| H | -0.03988700 | 2.98974600 | 0.41700300 |
| Sn | 1.08670400 | -1.88513900 | -0.15336100 |
| H | 2.54623200 | -1.78215100 | 0.68018800 |
| H | -0.03887500 | -2.98980000 | 0.41653800 |
| H | 1.21519800 | -1.71955600 | -1.82329800 |

## Supplementary References


1. Shen, Z. & Lu, X. Cupric halide-mediated intramolecular halocyclization of N-electron-withdrawing group-substituted 2-alkynylanilines for the synthesis of 3-haloindoles. *Adv. Synth. Catal.* **351**, 3107-3112 (2009).
2. Nesmeyanov, A. *et al.* Tris(triphenylphosphinegold)oxonium salts. *J. Organomet. Chem.* **201**, 343-349 (1980).
3. Moroni, M., Moigne, J. L., Pham, T. & Bigot, J.-Y. Rigid rod conjugated polymers for nonlinear optics. 3. Intramolecular H bond effects on poly(phenyleneethynylene) chains. *Macromolecules* **30**, 1964-1972 (1997).
4. Clentsmith, G. K., Field, L. D., Messerle, B. A., Shasha, A. & Turner, P. Intramolecular cyclization of ortho-alkynylanilines by Rh(I)-catalyzed hydroamination to yield benzo(dipyrroles). *Tetrahedron Lett.* **50**, 1469-1471 (2009).
5. Sheldrick, G. M. SHELXL-97 (Univ. Göttingen, 1997).
6. Atwood, J. L. & Barbour, L. J. Molecular graphics: from science to art. *Cryst. Growth Des.* **3**, 3-8 (2003).
7. Tao, J. M., Perdew, J. P., Staroverov, V. N. & Scuseria, G. E. Climbing the density functional ladder: Nonempirical meta-generalized gradient approximation designed for molecules and solids. *Phys. Rev. Lett.* **91**, 146401-1-146401-4 (2003).
8. Grimme, S., Antony, J., Ehrlich, S. & Krieg, H. A consistent and accurate ab initio parameterization of density functional dispersion correction (DFT-D) for the 94 elements H-Pu. *J. Chem. Phys.* **132**, 154104-1-154104-19 (2010).
9. Figgen, D., Rauhut, G., Dolg, M. & Stoll, H. Energy-consistent pseudopotentials for group 11 and 12 atoms: adjustment to multi-configuration Dirac–Hartree–Fock data. *Chem. Phys.* **311**, 227-244 (2005).
10. Stoll, H., Metz, B. & Dolg, M. Relativistic energy-consistent pseudopotentials—Recent developments. *J. Comput. Chem.* **23**, 767-778 (2002).
11. Hay, P. J. & Wadt, W. R. Ab initio effective core potentials for molecular calculations. Potentials for K to Au including the outermost core orbitals. *J. Chem. Phys.* **82**, 299-310 (1985).
12. Frisch, M. J. *et al. Gaussian 09*, Revision D.01 (Gaussian, Inc., Wallingford CT, 2013).
13. Adamo, C. V., Barone, V. Toward reliable density functional methods without adjustable parameters: The PBE0 model. *J. Chem. Phys.* **110**, 6158-6170 (1999).
